# On the Construct Validity of Performance-Based Emotion Recognition Tests: Correlations with Social-Emotional Functioning and Cognitive Skills

**DOI:** 10.5334/pb.1443

**Published:** 2026-05-25

**Authors:** Emalie Hendel, Marc Brysbaert

**Affiliations:** 1 The Centre for Advanced Research in Experimental and Applied Linguistics, Wilson Hall, McMaster University, Sterling St, Hamilton, ON L8S, Canada; 2 Department of Experimental Psychology, Ghent University, 9000 Gent, Belgium

**Keywords:** Emotional perception, emotional intelligence, crystallised intelligence, subjective ratings, validity

## Abstract

This study examined the construct validity of performance-based emotion recognition tests. We recruited 227 adults (30–60 years old) through Prolific to complete four emotion recognition tasks in addition to measures of self-reported empathy, crystallised intelligence, social confidence, loneliness/well-being, interest in people versus things, and reading enjoyment (all measured with at least two indicators). Consistent with previous research, performance-based emotion recognition tasks were positively correlated and formed a separate cluster. This cluster correlated with crystallised intelligence but not with self-reported emotion recognition skills, social-emotional functioning, interest in people vs. things, or reading pleasure. Overall, our findings suggest that performance-based emotion recognition tests primarily assess skills related to those measured by performance-based tests of cognitive intelligence, rather than skills related to the social-emotional functioning as experienced by the participants. This may partially explain the low correlation between subjective and performance-based measures of emotion recognition.

## Introduction

Emotional intelligence is defined as an individual’s capacity to accurately perceive and understand emotions, manage emotions to maintain a positive state, and use emotions to facilitate decision-making (Salovey & Mayer, 1990). These skills are fundamental to human social functioning. As a result, much research has been devoted to them, both in typical populations and in groups with impaired performance (Franca et al., 2023; Roberts et al., 2010; Schlegel et al., 2019).

A thorny issue in emotional intelligence research is the disagreement about how to measure the skills involved. The remainder of this article will focus on emotion perception, but the same issue exists for emotion management and using emotions to make good decisions. Emotion perception can be defined as “the ability to recognize, identify and decode emotional information in the environment through multiple emotional cues”, including facial expressions, prosody, body posture, and social context (Bornhofen & Mcdonald, 2008; in Heredia et al., 2022). Although these can be further distilled and tested as separate skills, we take emotion perception to mean both the recognition of emotions in images or videos as well as the understanding of emotional content in social contexts and written texts.

Still, the breadth of skills underlying emotion perception poses a problem for how it can be measured. A first approach consists of self-reports in which people are asked to indicate how good they are in various emotion-related situations. Typical items include “I can easily work out what another person might want to talk about” and “I am good at predicting what someone will do”, as asked in the Questionnaire of Cognitive and Affective Empathy (QCAE; Reniers et al., 2011). The second approach is performance-based and investigates how well people can recognize emotions in stimuli. For example, a short video is shown of someone expressing anger and participants are asked to say how the persons feels, as in the Geneva Emotion Recognition Test (GERT; Schlegel et al., 2014), or participants have to indicate how a person is likely to feel in the following situation: “Xavier completes a difficult task on time and under budget. Xavier is most likely to feel?”, included in the Situational Test of Emotion Understanding (STEU; MacCann & Roberts, 2008).

Unfortunately, the two measures do not correlate with each other, raising doubts as to whether they measure the same concept. A meta-analysis by Murphy and Lilienfeld (2019) of 85 studies (268 effect sizes, 14,327 participants) reported a correlation of only r = .10 between self-assessment scales and behavioral emotion recognition measures (1% shared variance). Heck et al. (2024) even reported a negative relationship between self-rated and objective social intelligence scores.

Murphy and Lilienfeld (2019) interpreted the low correlation between subjective and objective measures as evidence that participants lack knowledge of their emotional abilities and that self-assessments of emotion perception (in their case, understanding emotions) should be distrusted. In their own words (p. 1062): “These results raise serious concerns regarding the widespread use of self-report cognitive empathy scores as proxies for cognitive empathy ability, as well as the extensive theoretical conclusions that have been based on their use in past studies.” Low correlations between objective and subjective estimates of performance have been reported for other skills as well (e.g., Bommer et al., 1995; Crumley et al., 2014; Wendt et al., 2024). Even for intelligence, Zell and Krizan (2014) reported a correlation of only .29 between self-estimates and objective measures.

A potential issue with Murphy and Lilienfeld’s (2019) interpretation is that they provided little evidence for the construct validity of the performance-based tests, apart from face validity (the test seems to measure what it is intended to measure) and content validity (the task appears to cover relevant aspects of the intended concept). Put simply, it is therefore unclear whether the behavioural assessments which intend to evaluate emotion recognition ability truly measure this skill. This weakness becomes relevant when one considers that most of the studies with performance-based emotion recognition tests in Murphy and Lilienfeld’s meta-analysis made use of the popular Reading the Mind in the Eyes Test (RMET; Baron-Cohen et al., 2001). In the RMET, participants are shown photographs of people’s eyes and asked to indicate which emotion is being expressed out of four possible answers.

Despite its wide use, the RMET has been heavily criticized in recent years as a test of emotion perception (Beffara et al., 2025; Hafner et al., 2026; Higgins et al., 2024). It was originally proposed as a measure to test theory of mind (the ability to attribute mental states to oneself and others and to understand that the mental states of others may differ from one’s own), rather than a test of emotion perception (see, however, Kittel et al., 2022). Higgins et al. (2024) were most explicit in listing the problems with the construct validity of the RMET. They used six criteria: (1) test-retest reliability, (2) internal consistency, (3) factor structure, (4) convergent validity, (5) discriminant validity, and (6) known-other group validity. In a review of 1,461 articles with the RMET, they found that only 37% of the articles mentioned some validity evidence. The others relied solely on face validity and content validity. Worse still, the studies that examined one of the issues often failed to obtain convincing validity evidence (e.g., internal consistency was low or the test did not measure a single factor).

Murphy and Hall (2024) responded to Higgins et al. (2024) by stating that of the six criteria, convergent validity is the most important. They calculated that the RMET has a correlation of 0.4 with other performance-based tests for emotion recognition. A similar finding was reported by Franca et al. (2023), who found that the RMET had a correlation of 0.49 with a new performance-based test for emotion recognition that they had developed (the Seeing Emotions in the Eyes test, or SEE-48; see below). In the same vein, Schlegel et al. (2016) reported a correlation of .5 between the GERT, another test of emotion recognition, and a test of emotion understanding, the STEU. So, it looks like performance-based tests for emotion recognition and comprehension measure a shared skill, even if they do so imperfectly.

The next question is whether the common skill is the intended ability of emotion perception (discriminant validity). As MacCann and Roberts (2008, p. 541) pointed out: “Emotional intelligence tests should relate to variables or outcomes reasonably indicative of facility with emotions (e.g., coping with stress and lack of emotion-related disorders), demonstrating the appropriateness of the adjective emotion in emotional intelligence.” If a test of emotion perception measured the same skills as a vocabulary test or a general knowledge test (two commonly used tests of crystallised intelligence), most people would rightly object that the name raises false expectations (jangle fallacy; Hodson, 2021). On the other hand, if the test predicted who is emotionally happy, has good social relationships, can easily work with others, enjoys a job that involves constant interaction with people and is a good coach, most people would agree that this is a true test of emotional intelligence.

Developers of performance-based tests for emotion perception have struggled to provide strong evidence that their tests measure emotional functioning better than a cognitive IQ test (if they have provided such evidence at all). Schlegel et al. (2016) found not only correlations of 0.5 between the GERT and other emotion tests, but also correlations of 0.4 with vocabulary size and general reasoning ability. Similarly, MacCann and Roberts (2008) reported a correlation of 0.5 of the STEU with vocabulary. In a large-scale study involving multiple tests, MacCann et al. (2014) concluded that emotional intelligence tests not only correlate strongly with typical cognitive IQ tests but also load almost as strongly on general intelligence as typical cognitive factors such as fluid intelligence, crystallised intelligence and quantitative reasoning. The authors remained convinced of the usefulness of emotional intelligence tests as a separate factor of general intelligence, even though the high correlations suggest that it may be difficult to predict social-emotional functioning much better with a performance-based emotion perception test than with an existing IQ test.

Other pioneers in the field of performance-based emotional intelligence tests came to the same conclusion. Mayer et al. (2025) summarized their view of emotional intelligence as follows. For them, emotional intelligence is a broad intelligence form that is part of general intelligence. It is on the same level as visual-spatial, mathematical and verbal-propositional intelligences, with which it has correlations of .6 (corrected for measurement error). Nevertheless, emotional intelligence is assumed to have incremental validity over other broad intelligences in predicting important outcomes such as academic performance, work performance, the quality of social relationships and psychological health.

Since performance-based tests of emotion perception are strongly related to general cognitive intelligence, we may have to rethink Murphy and Lilienfeld’s (2019) explanation for the weak link between self-reported and performance-based measures of emotional intelligence. Is it possible that the low correlation stems from performance tests largely measuring general intelligence instead of social-emotional abilities?

The first objective of the current study was to verify whether performance-based tests of emotion recognition measure a single factor (convergent validity). To do so, we included four performance-based tests of emotion recognition. The first one was the SEE-48 test developed by Franca et al. (2023). Similar to the RMET, participants in this test are asked to identify the emotion expressed in the eyes of actors. The test improves upon the RMET by having higher internal consistency, measuring a single factor, and limiting the vocabulary to an absolute minimum by restricting the emotions to happiness, sadness, fear, anger, disgust, and surprise. The SEE-48 test correlates .46 with the STEU. It therefore measures a broader skill than just seeing emotions in the eyes.

In addition to the SEE-48, three other performance-based tests for emotion recognition and comprehension were included in the study: MRMET, GERT and STEU. MRMET is another attempt to improve the RMET (Kim et al., 2022). It has the same format as the RMET, but has a higher internal consistency, a more convincing factor structure, is less demanding verbally, and does not contain items that may raise questions about gender or race stereotypes. The GERT was added to make sure that emotion perception was not limited to seeing emotions in eyes. This test includes short clips of people interacting (including audio). The STEU even goes beyond simple emotion perception and has been proposed as a test of emotion understanding. Participants must indicate how people are likely to feel in a variety of situations.

The second objective of this study was to determine how the cluster of performance-based emotion recognition tests (if found) relates to other factors. We included two tests of crystallised intelligence (vocabulary and general knowledge) to see how much performance-based emotion recognition tests differ from them (discriminant validity). We further included questionnaires of self-reported emotion recognition to verify Murphy and Lilienfeld’s (2019) observation of a low correlation between these two measures. Finally, we included three criteria, which have been proposed as outcomes of good emotion perception (criterion validity): self-perceived social-emotional functioning, greater interest in jobs that require a lot of interaction with people, and interest in reading fiction for pleasure. The second criterion was included because it seems logical that people with low emotional intelligence would avoid occupations that require constant interaction with others. Interest in people is a characteristic on which women score higher than men (Archer, 2019; Kuhn & Wolter, 2022), and Greenberg et al. (2023) used women’s higher scores on the RMET as group-based evidence for the validity of the RMET. The third criterion was added because several correlations have been reported between emotional intelligence and fiction reading (Castano, 2024; Eekhof & Mar, 2023), either because reading fiction makes people better at understanding emotions (Mar et al., 2009) or because people who are good at understanding emotions enjoy reading fiction more than people who are less good at it (Samur et al., 2017).

All in all, there were six constructs: performance-based emotion perception, crystallised intelligence, self-reported emotional intelligence, self-reported socio-emotional functioning, interest in jobs that involve interactions with people, and reading pleasure. Each construct was measured with at least two tests to make sure that a negative finding was not due to an unreliable test or a test without convergent validity (Brysbaert, 2024; Hodson, 2021; Völker, 2020).

## Method

### Participants

This study was run through the online data collection service Prolific (https://www.prolific.co/) in October 2023. The study lasted approximately an hour and a half, and each participant received £18.00 for completing the study. Since we anticipated finding small correlations nearing r = .2, we followed the recommendation of Vermeiren et al. (2023) and tested upwards of 200 participants.^1^ Of the 250 participants who completed the study, *n* = 21 had missing data on at least one measure and were excluded from our analyses. One individual identified as non-binary and one person did not identify their gender. Because of this low representation of gender identities other than women and men, we removed these two participants from our analyses. This left us with a total of 227 participants with complete data.

Because we wanted our sample to be different from a student population, we limited the age range to 30–60 years. Older participants were not included because some performance drop may be observed on some tests for them (Connolly et al., 2021). On average, participants were 40.8 years old (SD = 8.33), with 111 identifying as women and 116 identifying as men. There was no correlation between age and gender. All participants had English as their first language, no language related disorders and no mild cognitive impairments or dementia, and a Prolific approval rate between 90 and 100 on prior submissions.

### Materials

We included 21 tests in our study. A brief description is reported here. For additional details, please refer to the Materials section of the Supplementary Files.

#### Test-based Emotion Recognition and Understanding

**Geneva Emotion Recognition Test short version (GERT-S)**. We used the short, 10-minute version of the GERT to test participants’ ability to recognize emotions when observing 42 short video clips of actors expressing emotions (Schlegel et al., 2014, 2019; Schlegel & Scherer, 2016). Participants selected one out of 14 possible responses for each video clip, only one of which was correct.

**Situational Test of Emotional Understanding (STEU)**. We used the STEU (MacCann & Roberts, 2008) to measure the understanding of relationships between emotions and verbally described circumstances. We used only the 25 items that had the best psychometric properties in previous studies with published raw data (see OSF repository). Participants were asked to choose among five multiple-choice response options, only one of which was correct. An example of an item is “An irritating neighbour of Eve’s moves to another state. Eve is most likely to feel __: regret, hope, relief, sadness, joy.”

**Seeing Emotions in the Eyes test (SEE-48)**. We used the new SEE-48 test (Franca et al., 2023), which shows participants 48 photos of actors expressing one of Ekman’s six primary emotions: anger, disgust, fear, surprise, happiness, and sadness (eight photos per emotion). Only the eyes region is shown, and participants must choose the correct emotion among the six primary emotion alternatives given.

**Multiracial Reading the Mind in the Eyes Test (MRMET)**. Given the problems with the RMET, we used a recently published, multiracial alternative (Kim et al., 2022). It has the same format as the RMET, but has a higher internal consistency, a more convincing factor structure, is less demanding verbally, and does not contain items that may raise questions about gender or race stereotypes. Participants are shown the eyes of actors expressing a multitude of emotions and must select the correct response out of four options. There are 37 items from professional actors of different ages, genders, and races.

#### Self-evaluation of emotion perception ability

**Questionnaire of Cognitive and Affective Empathy (QCAE)**. The QCAE is a test of emotion perception (Reniers et al., 2011). It measures five factors. The first two are related to cognitive empathy, or the ability to create a mental representation of another person’s cognitive and emotional states within one’s own mind. They are: perspective taking (“I can tell if someone is masking their true emotion”) and online simulation (“I try to look at everyone’s side of a disagreement before I make a decision”). The last three factors measure affective empathy, or the ability to quickly recognize someone’s emotions based on various cues and to elicit a corresponding emotional response. They are: emotion contagion (“I am happy when I am with a cheerful group and sad when the others are glum”), proximal responsivity (“I often get emotionally involved with my friends’ problems”), and peripheral responsivity (“I am usually objective when I watch a film”). Participants rated all items on a four-point Likert scale ranging from “strongly agree” to “strongly disagree”. Items that were negatively phrased, were reverse coded (see Reniers et al., 2011).

**Metacognition of emotion perception**. We added 16 items to measure participants’ metacognitive judgments about their ability to recognize another person’s emotions (see Table S1 in Supplementary Files). Participants were asked whether they thought they were good at identifying eight different emotions in the eyes or face of other people (e.g., “It is hard for me to tell from someone’s eyes if they are angry” and “It is hard for me to tell from someone’s face if they are happy”). The questions were added to the QCAE items before randomizing all items. They were negatively worded to counteract the acquiescence bias that might be introduced by the predominantly positively formulated items of the QCAE and as an attention check for careless responding.

#### Crystallised intelligence

**Student Vocabulary Test version 4 (StuVoc4)**. We created a new vocabulary test, called StuVoc4, based on previous work by Vermeiren et al. (2023). We combined the odd-numbered items from StuVoc1 with the even-numbered items from StuVoc3 to create a 50-item vocabulary test (see Supplementary Files Table S2 for items) in which participants had to choose the correct answer among four response options. The new test was created in case we wanted to repeat the study with participants who have English as a second language.

**General Knowledge test (GK)**. We administered the GK (Vermeiren et al., 2023) as a second measure of participants’ crystallised intelligence. This test contains 40 questions about a wide range of topics (e.g., “Why is it imperative that astronauts work out in space”, “What does al dente mean in cooking”), with one correct answer out of four possible options.

#### Self-perceived social-emotional functioning

**Lubben Social Network Scale (LSNS-6)**. We used the LSNS-6 (Lubben et al., 2006) to measure the number and closeness of relationships between participants and their family members and friends. Participants responded to three questions about family (“How many relatives do you see or hear from at least once a month?”) and three questions pertaining to friendships (“How many friends do you feel close to such that you could call on them for help?”) on a five-point scale ranging from “0 = none” to “5 = nine or more”.

**Satisfaction With Life Scale (SWLS)**. High emotional intelligence is expected to lead to high well-being. So, we asked participants to rate five items pertaining to their satisfaction with life (Diener et al., 1985), on a 7-point scale ranging from one “strongly disagree” to seven “strongly agree”.

**Social Efficacy**. We included five questions about the participants’ perceived capacity to manage various interpersonal situations (Caprara & Steca, 2005; Di Giunta et al., 2010; Irwing et al., 2023): “How well can you express your opinion to people who are talking about something of interest to you?”, “How well can you work or study with others?”, “How well can you help someone new become part of a group to which you belong?”, “How well can you share an interesting experience you had with other people?”, and “How well can you actively participate in group activities?”. Participants gave ratings on a 5-point Likert scale ranging from one “not well at all” to five “very well”.

**Social Confidence**. We included six items to measure perceived “propensity to feel confident in social situations” (Irwing et al., 2023): “In social situations, I generally make the first move”, “I am apprehensive about new encounters (reverse coded)”, “I am good at making unprepared speeches”, “I am not good at taking charge of a group (reverse coded)”, “I feel comfortable around people”, and “I am confident that I can handle challenging social situations”. Participants gave ratings from one “Strongly Disagree” to seven “Strongly Agree”, and negatively worded items were reverse coded.

**Social Curiosity Scale (SCS)**. Interest in other people’s thoughts, behaviours, and feelings has been linked to social competence, personality traits, and social anxiety. To measure this trait, we used Renner’s (2006) 10-item SCS. Participants used four-point Likert scales to rate their own general social curiosity, pertaining to a broad interest learning about people (‘‘When I meet a new person, I am interested in learning more about him/her.’’), and covert social curiosity, pertaining to inconspicuously fulfilled social curiosity (“When on the train, I like listening to other people’s conversations.”).

**UCLA Loneliness Scale Version 3 (UCLA)**. The UCLA measures participants’ subjective feelings of loneliness and isolation (Russell, 1996). Participants rated 20 positively and negatively worded statements related to loneliness and social relationships on a four-point Likert scale ranging from one “never” to four “often”. Negatively worded items were reverse coded.

**Adult Rejection Sensitivity Questionnaire (A-RSQ)**. Rejection sensitivity is a person’s ability to quickly detect and react to any incoming threat of social rejection. This has been linked to maladaptive patterns within social relationships (Berenson et al., 2009). We included nine items from two subscales from the A-RSQ (Berenson et al., 2009; Downey & Feldman, 1996) to measure this construct: the concern or anxiety they would feel faced with each situation (i.e., rejection concern), and the perceived likelihood of being rejected within each one (i.e., rejection expectancy). Participants rated each item twice, on a Likert scale ranging from one “very unconcerned/very unlikely” to six “very concerned/very likely”.

#### Interest in people vs. things

**Occupations Questionnaire**. To find out to what extent participants preferred to work with people than with things, we created a questionnaire in which participants were asked which of each pair of occupations they would most like to pursue or receive training for. Each pair contained one things-oriented job and one person-oriented job which were matched on social status, based on data collected by Lippa et al. (2014). Examples are “Astronautic engineer vs. Physician” and “Roofer vs. Waiter.” We alternated the left and right presentation of the things-oriented and person-oriented jobs randomly. Scores on this measure were the sums of the item responses to each category. Items are presented in the Supplementary Files Table S3.

**Personal Globe Inventory – Short (PGI-Short)**. We included the PGI-Short (Tracey, 2010) to measure participants’ vocational interests as a function of interest in people vs. things, data vs. ideas, and levels of prestige (Tracey, 2002). Participants responded to each of the 40 items twice, rating the degree to which they liked doing the given activity and their degree of perceived competence for each activity (Tracey, 2010) on a scale from one “Strongly dislike/Unable to do” to seven “Strongly like/Very competent”. We computed the preference for jobs with people according to the key given in the appendix (Key 21).

#### Reading pleasure

**Reading Habits Questionnaire (RHQ)**. We used the RHQ (Kuijpers et al., 2020) to measure the frequency with which participants read 21 text genres. Participants rated how often they read each of the various genres of fiction, nonfiction, and popular media sources from zero “not at all” to six “extremely”. This gave us two individual subscales to include in our analyses: fiction reading and nonfiction reading. The items on social media and emails were not included, as it is not clear to which category they belong.

**Author Recognition Test (ART3)**. We used Vermeiren et al.’s (2023) updated version of the ART (Acheson et al., 2008; Vermeiren et al., 2023) as an objective measure of participants’ exposure to fiction. Participants had to decide whether each one of 90 names referred to real authors. Of these, 60 names did belong to real authors, while 30 did not. Wrong author answers were penalised, with the final score calculated as the percentage of authors recognised minus the percentage of yes-answers given to non-authors.

**Predictors of Leisure Reading (PoLR) scale**. This scale includes three factors related to reading for pleasure: reading motivators, reading demotivators, and reading attitudes (Martin-Chang et al., 2021). There are five items related to reading motivation (“I read for entertainment”), five items related to reading demotivation (“I often don’t read because I find it boring”), and six items related to reading attitudes (“I think of myself as a reader”). Participants gave their responses on a seven-point scale ranging from “Disagree strongly” to “Agree strongly”.

**Modified Tellegen Absorption Scale (MODTAS)**. To measure participants’ absorption in a reading experience (Jamieson, 2005; Tellegen & Atkinson, 1974; Terhune & Jamieson, 2021), we used the MODTAS (Jamieson, 2005). Participants rated 34 items which examined their predispositions to experiencing imaginative and perceptual states (“I imagine some things so vividly that they hold my attention as good as a movie or story does”, “When I listen to music, I get so caught up in it that I don’t notice anything else”). Participants answered on a Likert scale ranging from zero “never” to four “very often”.

### Procedure

Participants were recruited via Prolific and informed that they would be asked to complete several measures on recognizing emotions in people’s eyes, understanding emotions, and reading. To motivate participants, they were told that they would receive feedback on their performance on some emotion recognition measures at the end of the study. Participants were first asked to give some demographic information, including age, gender, highest level of education, first language, and job status. They then began the test battery. To reduce response biases, tests which measured similar concepts (e.g., loneliness, social confidence, interest in things vs. people) were divided so that some were presented in the beginning of the questionnaire, and some were presented at the end. The questionnaire began with the MODTAS, PGI-Short, SCS, UCLA, Social Efficacy, and Social Confidence. Next, participants completed all four measures of emotion recognition and understanding, starting with the SEE-48, STEU, MRMET, and GERT-S. Participants then completed the Occupations Questionnaire, QCAE and Metacognition questions, SWLS, LSNS-6, and A-RSQ. Finally, participants completed the PoLR, ART3, RHQ, StuVoc4, and GK. Once the questionnaire was complete, participants were given the percentage of faces they had accurately recognized in the SEE-48 and the GERT-S before being redirected to the Prolific website and receiving payment for their participation. All data and code for the analyses reported in this manuscript are available at https://osf.io/kcqe4/.

## Results

### Data Processing and Descriptive Statistics

Descriptive statistics and reliability of the tests are presented in Table 1. For the performance-based tests, we coded each of the correct answers as 1 and incorrect answers as 0. For the self-rating measures, we used the Likert scores. Where a test had negatively phrased items (e.g., in the UCLA, Social Confidence, QCAE, or PoLR), we used reversed scoring so that the highest ratings became the lowest and vice versa.

**Table 1:** Descriptive Statistics.


TEST	WOMEN (N = 111)		MEN (N = 116)		d	OVERALL (N = 227)		ALPHA	OmegaH	OmegaT
		
	M	SD	M	SD		M	SD			

Age (Years)	40.91	8.82	40.63	7.87		40.77	8.33			

Highest Level of Education^1^										

*Bachelor’s degree or equivalent undergraduate qualification*	47 (42%)		56 (48%)			103 (45%)				

*Doctoral degree or equivalent advanced qualification*	2 (2%)		2 (2%)			4 (2%)				

*Master’s degree or equivalent postgraduate qualification*	20 (18%)		20 (17%)			40 (18%)				

*Secondary education (high school or equivalent)*	42 (38%)		38 (33%)			80 (35%)				

Absorption (MODTAS)	1.61	0.73	1.52	0.64	0.13	1.56	0.69	0.95	0.79	0.95

Vocational Interests (PGI-Short)										

*Liking_jobs_people*	1.28	0.95	0.23	1.03	1.06	0.74	1.12	- -	- -	- -

*Compentence_jobs_people*	1.39	0.88	0.45	0.94	1.02	0.91	1.02	- -	- -	- -

*General Social Curiosity*	3.06	0.47	2.94	0.57	0.22	3.00	0.53	0.87	0.82	0.92

*Covert Social Curiosity*	2.81	0.59	2.36	0.68	0.71	2.58	0.67	0.85	0.73	0.90

Loneliness (UCLA)	2.31	0.61	2.37	0.66	–0.09	2.34	0.64	0.95	0.78	0.96

Social Efficacy	3.91	0.66	3.93	0.73	–0.03	3.92	0.70	0.82	0.77	0.86

Social Confidence	3.76	1.20	3.93	1.32	–0.13	3.84	1.26	0.85	0.75	0.90

SEE-48	0.63	0.11	0.64	0.12	–0.14	0.63	0.11	0.69	0.67	0.71

STEU	0.71	0.12	0.68	0.13	0.24	0.70	0.12	0.59	0.18	0.62

MRMET	0.71	0.11	0.67	0.11	0.39	0.69	0.11	0.64	0.16	0.67

GERT	0.57	0.12	0.55	0.13	0.16	0.56	0.13	0.71	0.40	0.73

Occupations Questionnaire	–0.40	0.17	–.59	0.19	1.07	–0.50	0.20	0.84	0.55	0.86

Empathy (QCAE)										

*Perspective taking*	2.87	0.50	2.82	0.47	0.11	2.84	0.47	0.87	0.65	0.90

*Online simulation*	2.99	0.49	2.98	0.55	0.01	2.98	0.52	0.86	0.83	0.90

*Emotion contagion*	2.90	0.60	2.73	0.67	0.27	2.81	0.64	0.78	0.70	0.83

*Proximal responsivity*	3.00	0.53	2.78	0.67	0.37	2.89	0.61	0.74	0.66	0.78

*Peripheral responsivity*	2.97	0.56	2.68	0.62	0.49	2.82	0.61	0.69	0.09	0.74

Metacognition	3.19	0.53	3.10	0.56	0.16	3.14	0.55	0.95	0.85	0.97

Life Satisfaction (SWLS)	4.17	1.44	3.93	1.53	0.16	4.05	1.49	0.92	0.85	0.94

Lubben Social Network (LSNS-6)	2.55	0.89	2.33	0.92	0.24	2.44	0.91	0.83	0.57	0.93

Rejection Sensitivity (A-RSQ)										

*Rejection Concern*	3.50	0.96	3.57	1.00	–0.07	3.53	0.98	0.84	0.68	0.87

*Rejection Expectancy*	2.41	0.63	2.59	0.76	–0.26	2.50	0.00	0.77	0.57	0.83

Predictors of Leisure Reading (PoLR)										

*Reading Motivation*	5.59	1.16	5.08	1.38	0.40	5.33	1.30	0.91	0.82	0.94

*Reading Demotivation*	2.32	1.44	2.88	1.59	–0.37	2.61	1.54	0.92	0.89	0.93

*Reading Attitudes*	5.41	0.78	5.23	0.98	0.21	5.32	0.89	0.74	0.56	0.84

Author Recognition (ART3)	0.38	0.21	0.32	0.21	0.25	0.35	0.21	- -	- -	- -

Reading Habits (RHQ)										

*Fiction*	1.86	1.08	1.69	1.14	0.15	1.77	1.11	0.88	0.64	0.91

*Nonfiction*	2.04	1.23	2.45	1.30	–0.32	2.25	1.28	0.78	0.66	0.87

Vocabulary (StuVoc4)	0.86	0.13	0.85	0.14	0.03	0.85	0.13	0.91	0.56	0.92

General Knowledge (GK)	0.71	0.13	0.75	0.13	–0.32	0.73	0.13	0.79	0.36	0.81


*Note*. This table presents the means and SD for each measure. Effect size differences between genders are represented by Cohen’s d, with positive values indicating an advantage for women, and negative values indicating an advantage for men. Reliability statistics include alpha, omega hierarchical (OmegaH), and omega total (OmegaT). These could not be calculated for tests involving a difference score (indicated by - - in the table). ^1^Highest Level of Education is presented in terms of total *n* for each value, with the percentage of participants in parentheses.

### Correlation matrix

Each validation analysis is an interpretation of the correlations between the tests. Therefore, the correlation matrix is the most informative, even though in this case it is a fairly large matrix (Excel files can be found in the osf repository). An analysis of the skewness and kurtosis values indicated that the data distributions were approximately normal (most extreme skewness value = –1.4, most extreme kurtosis value = 1.7). Nevertheless, we present both Pearson correlations (above the diagonal) and Spearman correlations (below the diagonal) in the full correlation matrix (Figure 1 in Supplementary Materials).

To bring some order to the correlation matrix, the variables were ordered using the hclust option in the R package corrplot (Wei & Simko, 2010). This uses a hierarchical cluster algorithm to group the variables with the highest correlations. The variables were scaled for the hclust analysis.

There were three main clusters of tests in the correlation matrix. The first included tests of self-perceived social-emotional functioning, reading and self-reported empathy. The second included performance-based tests for emotion recognition/understanding and tests for crystallised intelligence. The third related to a preference for jobs involving working with people. It is further important to note that there were virtually no positive correlations between clusters 1 and 2. The strongest positive correlation between these clusters was between reading motivation and scores on the author recognition test (ART; *r* = .32). There were even negative correlations between scores on the GERT test and social efficacy (*r* = –.20) and self-confidence (*r* = –.30). There were also virtually no correlations between clusters 2 and 3, with the sole exception of MRMET, which appeared to have some positive correlations with covert curiosity (*r* = .30) and competence in jobs involving people (*r* = .17).

### Exploratory Graph Analysis

To bring further structure in the pattern of correlations, we used exploratory graph analysis (EGA). This approach determines which observed measures are partially correlated with one another in such a way as to be clustered together within an interconnected network (Golino & Epskamp, 2017). The clusters that are identified give a good indication of the possible latent variables in the dataset (Golino & Epskamp, 2017; Goring et al., 2021). Therefore, using EGA was a first step in figuring out which of our tests measured similar constructs.

**Figure 1:**
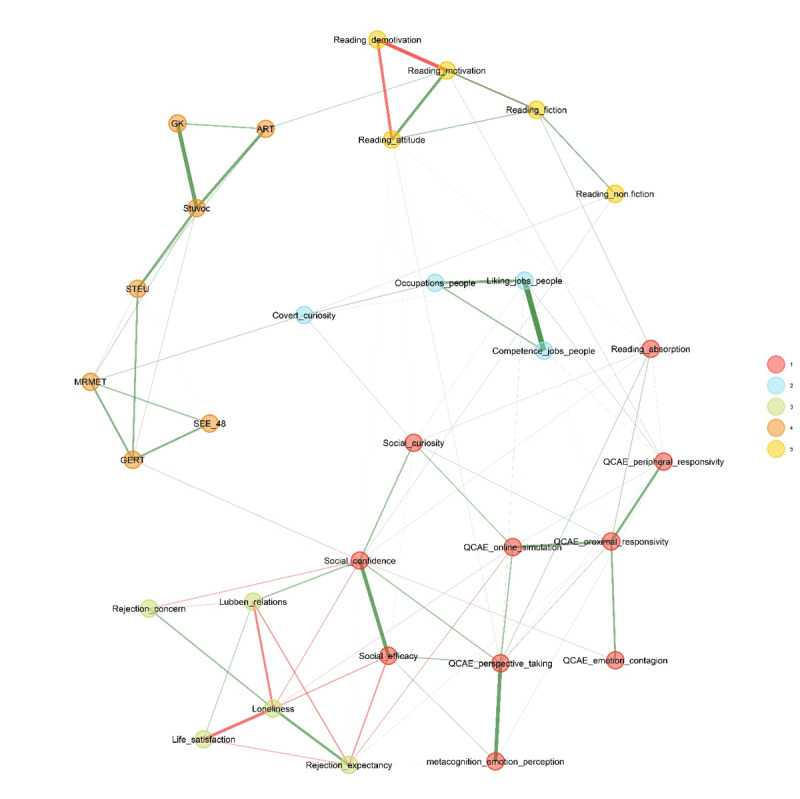
Exploratory Graph Analysis. *Note*. This graph shows the relationships between all 31 test and subtest scores included in our test battery.

We ran an EGA using the EGAnet [2.0.8] package (Golino & Epskamp, 2017) with the default values recommended by the authors (Model: GLASSO; EBIC with gamma = 0.5; Correlations: auto; Lambda: 0.22009187748298; n = 100; ratio = 0.1, community structure detection algorithm: walktrap). The five clusters which emerged from this analysis can be seen in Figure 1. This model included 88 edges with a density of 0.19, and a Total Entropy Fit Index (TEFI; Golino et al., 2021) of –37.61.

The first cluster mainly consisted of the *self-reported empathy* and the *self-reported ratings of social functioning*. The second cluster mainly involved *interest in jobs with people*. The third cluster corresponded to *well-being*, involving life satisfaction, social relationships, and negatively related to loneliness and rejection concerns. The fourth cluster involved the *performance-based emotion recognition tests* and the tests of *crystallised intelligence*. The Author Recognition Test also belongs to this cluster, as observed before (Vermeiren et al., 2023), suggesting that knowledge of authors is part of general culture and not mostly due to having read those authors. The fifth and final cluster was *reading pleasure*.

**Figure 2:**
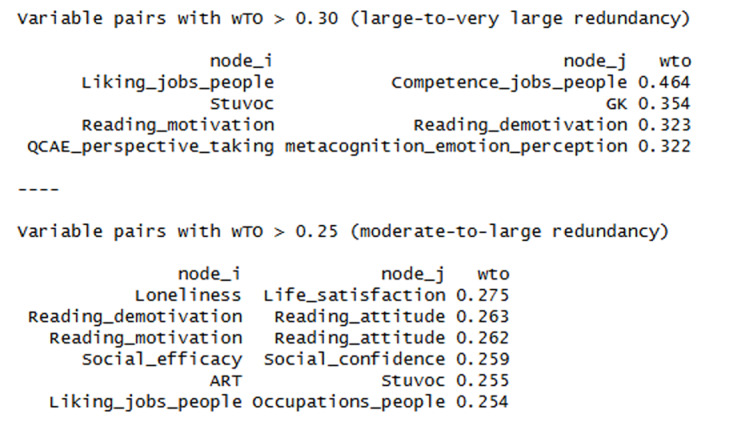
Test with too large redundancy given the overall network of tests examined.

A potential issue with the above analysis is that it may be influenced by redundant tests. These are tests that have a higher correlation than expected based on the network (these are the test pairs with high correlations in Supplementary Materials Figure 1 and thick edges in Figure 1). Christensen et al. (2023) developed an algorithm to detect local dependencies in graph networks. When this algorithm was applied, 10 test pairs were marked as redundant pairs. Figure 2 shows them.

**Figure 3:**
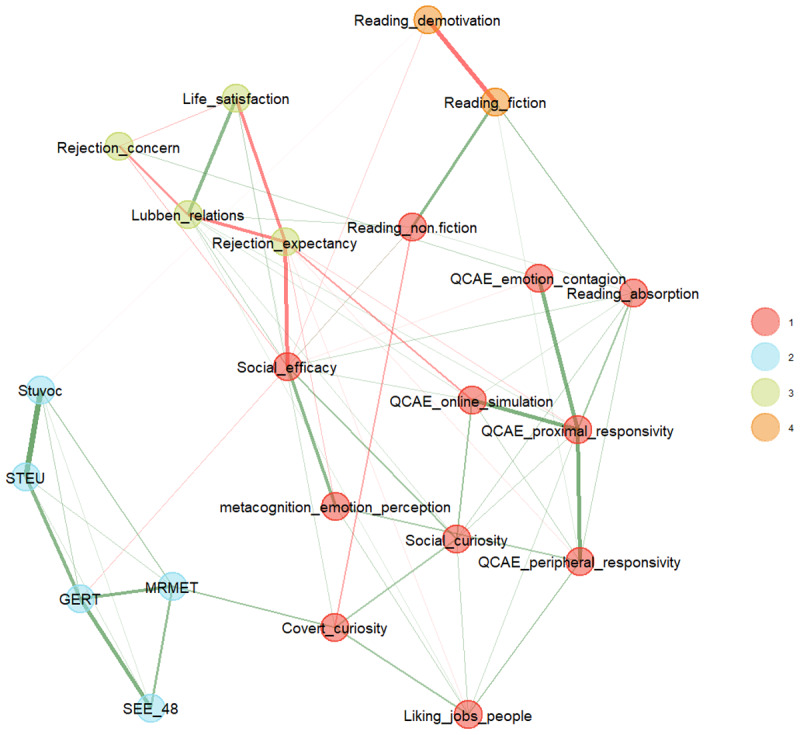
The EGA analysis when redundant items are omitted.

For each redundant test pair, the EGAnet package selects the test that has the strongest relations within the network and deletes the other test. The outcome of this analysis is shown in Figure 3. The resulting network had 65 edges with a density of 0.28, and a TEFI of –19.49. The structure remained very much the same, except that there was no longer enough evidence to distinguish between the cluster of self-rated empathy and liking jobs that involve interactions with people (mainly because the tests of the latter cluster all largely measured the same construct in the same way).

Bootstrapping can be used to see how stable the clusters are when new datasets are generated on the basis of the existing data and the EGA analysis repeated (Christensen & Golino, 2021). Table 2 lists the outcome of a parametric bootstrap with 500 replications, where item stability values larger than 0.70 indicate acceptable stability (Christensen & Golino, 2021). This analysis showed that the clusters of performance-based tests, well-being and reading preference were stable, but that the big cluster of self-ratings showed inconsistency, suggesting that some tests did not fully belong to the cluster (e.g., covert social curiosity) or belonged to two different clusters (e.g., social efficacy, which seems to load both on the cluster of well-being and on the cluster of self-ratings).

**Table 2:** Allocation to clusters in parametric bootstrapping with 500 replications.


TEST	CLUSTER				

	C1	C2	C3	C4	C5

QCAE_peripheral_responsivity	0.92				0.07

QCAE_proximal_responsivity	0.90				0.08

QCAE_online_simulation	0.87				0.09

QCAE_emotion_contagion	0.85				0.09

Social_curiosity	0.76		0.07		0.13

Liking_jobs_people	0.74		0.05	0.05	0.15

Reading_absorption	0.71			0.18	0.07

metacognition_emotion_perception	0.45		0.37		0.16

Covert_curiosity	0.42	0.09	0.07	0.16	0.21

Social_efficacy	0.24		0.58		0.15

Reading_non.fiction	0.14			0.67	0.11

Rejection_concern	0.05		0.83		0.10

SEE_48		1.00			

MRMET		1.00			

GERT		1.00			

STEU		0.99			

Stuvoc		0.99			

Life_satisfaction			0.89		0.08

Lubben_relations			0.89		0.08

Rejection_expectancy			0.85		0.10

Reading_demotivation				0.95	

Reading_fiction				0.95	


### Structural Equation Modeling

Although EGA provides us with a good starting point for interpreting the correlations between the different tests, it is limited because it does not give information about the correlations between the clusters, nor about the causes of the inconsistencies in the bootstrapping. Therefore, we translated the EGA networks from Figures 1 and 3 into structural equation models (SEMs), just like Vermeiren et al. (2023) did. The advantage of the SEM was that it allowed us to investigate how much each test contributed to the latent structure underlying our data while also considering the correlations between latent variables.

**Figure 4:**
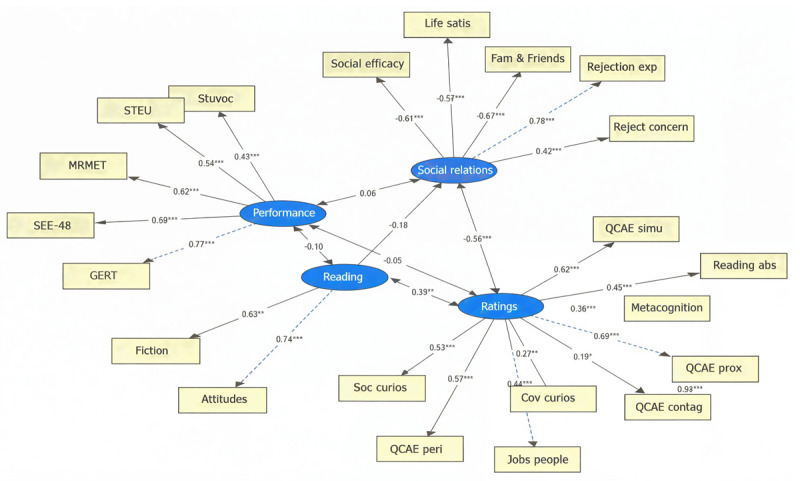
Structural Equation Model based on the EGA outcome shown in Figure 3. *Note*. This graph represents standardized coefficients between all measures and latent variables. Asterisks represent significance (*p* < .05, .01, and .001) for regression paths, latent paths, and covariances. Note that names of the measures have been abbreviated (e.g., Lubben_relations is now *Fam & Friends*).

Figure 4 shows the SEM equivalent of Figure 3. We used the R package lavaan [0.6.20] (Rosseel, 2012) with the robust maximum likelihood method. Analysis of the results and modification indices suggested some small changes that improved the fit of the model. We chose the minimal number of model respecifications and only implemented those which were theoretically plausible. First, we replaced Reading_demotivation with Reading_attitude, since the latter is defined as “a set of acquired feelings that consistently predispose an individual to engage in or avoid reading” (Conradi et al., 2014, p.154 in Martin-Chang et al., 2021), and so encapsulates both reading motivation and demotivation. We also dropped Reading_non.fiction, which did not significantly load on any of the factors. Since Social_efficacy pertains more to socioemotional functioning and relationships than it does to empathy ability, we allowed it to load on the factor *Social relations* (instead of [empathy] *Ratings*). Finally, we added residual correlations between QCAE_proximal_responsivity and QCAE_emotion_contagion (which both address empathetic involvement in others’ emotions), and between Stuvoc and STEU (which both rely on linguistic knowledge of the participant). The overall fit of the resulting model was SRMR = .08; RMSEA = .07; CFI = .81; TLI = .78. We used Marcoulides and Yuan’s (2017) equivalence testing method to assess the fit of our model. This revealed with 95% confidence that the population CFI is above 0.70 and that the size of misspecification (RMSEA) is no more than 0.081. Our reported model fit values (SRMR = .08; RMSEA = .07; CFI = .81; TLI = .78) fall above and below these adjusted fit values, respectively. Together, these adjusted indices indicate a fair model fit, although there is clearly room for improvement.

Overall, the SEM analysis shows the same four-cluster structure as in the EGA with non-redundant tests. The most important part of our analysis is the observation that the performance-based tests did not correlate with the ratings of empathy, with reading or with self-perceived social relations and well-being, as was already suggested by the correlation matrix in Supplementary Materials Figure 1. In contrast, there were significant correlations between self-rated empathy on the one hand and reading and self-perceived social-emotional functioning on the other hand.

## Discussion

The current study was designed to assess the extent to which performance-based tests of emotion perception differ from vocabulary and general knowledge tests, and to evaluate the predictive value of these tests for socio-emotional functioning. Participants were presented with four performance-based tests of emotion perception, as well as tests of crystallised intelligence and questionnaires measuring self-perceived social-emotional functioning, reading pleasure and interest in professions involving frequent interaction with others. Participants were also asked to rate the ease with which they could recognise emotions, as a subjective measure of emotion perception.

Our results confirmed that performance-based tests of emotion perception form a cluster of correlated tests (see Figures 2, 3, 4) and thus measure a common set of skills (Murphy & Hall, 2024). In line with Murphy and Lilienfeld (2019), this cluster did not correlate with subjective measures of emotion perception. However, the cluster also failed to correlate with the three validation criteria: self-perceived social-emotional functioning, interest in jobs that require frequent interaction with people and interest in reading fiction for pleasure. Therefore, performance-based tests designed specifically to measure emotion perception did not predict the benefits commonly associated with good emotion recognition. In fact, there were some indications of a negative correlation, particularly with the GERT. This suggests that people who scored highly on performance-based emotion perception tests tended to be less socially confident than those who scored low. This is in line with the hypersensitivity hypothesis, which states that paying too much attention to other people’s reactions is not always beneficial in social interactions (Nicolet-dit-Félix et al., 2023). Similar negative correlations between self-rated social skills and performance-based emotion recognition tests have been reported by Brown et al. (2023) and Heck et al. (2024).

We can think of three possible reasons why there is no correlation between the performance-based tests of emotion recognition and the validation criteria that we employed. Firstly, all the validation criteria were based on self-reports, as this was the only way we could test enough people given the resources available to us. If participants lack insight into their level of social functioning, it is unrealistic to expect a correlation between performance-based tests of emotion recognition and self-reported social functioning. One challenge with this interpretation is that it assumes people have limited insight into their own social and emotional functioning. So, people who say they feel lonely may not ‘actually’ be lonely, and people who say they are interested in books may not ‘actually’ read more books than those who say they are not interested in books.

This interpretation also predicts that performance-based tests of emotion recognition will correlate with performance-based indications of socio-emotional functioning, such as the number of people with whom individuals effectively interact, their performance in socially demanding environments or how they are perceived by others. An interesting study in this respect was published by McLean et al. (2026). They developed a performance-based emotion recognition test for police officers using body-cam videos of interactions between officers and citizens. To validate the test, the authors correlated the officers’ test scores with the number of force-related incidents over 18 months. They found a significant negative correlation between scores on the emotion recognition test and the use of force, suggesting that better emotion recognition reduces the likelihood of force being used during interventions. While this is encouraging, the correlation was no longer significant when the authors controlled for shift assignment and precinct, suggesting that the correlation could be due to a preference for day shifts and less demanding precincts. Furthermore, McLean et al. (2026) did not include a cognitive intelligence test, which is likely to have correlated with their emotion recognition test.

A second reason for the low correlation between performance-based emotion recognition tests and the validation criteria may be that the tests measure skills other than the perception of emotions. Performance-based tests are, by definition, challenging. Accuracy is the dependent variable, so the stimuli must contain minimal information (e.g. only the eyes are shown, or short, isolated stimuli are used). Most interactions with people are likely to be more explicit, meaning small differences in perceptual identification have little value in everyday social life. Such differences may reveal more about a person’s astuteness than how they choose to respond in social situations.

Whereas the cluster of performance-based emotion recognition tests did not correlate with social-emotional functioning, it did correlate strongly with crystallised intelligence tests, such as vocabulary and general knowledge tests, as well as knowledge of fiction authors. While such correlations have been reported before (MacCann et al., 2014; Mayer et al., 2025; Talipski et al., 2026), to our knowledge, this is the first study to simultaneously demonstrates a high correlation with intelligence tests and an absence of correlations with social-emotional validation criteria. Indeed, the correlations with crystallised intelligence tests were so high that several analyses failed to distinguish clearly between the clusters of performance-based emotion recognition and the crystallised intelligence tests. Such a distinction is likely to be found with a wider set of tests, as MacCann et al. (2014) and Mayer et al. (2025) argued, but the correlation between the two clusters will stay high, potentially of the same magnitude as that between the factors currently distinguished in analytical intelligence (e.g. the 16 broad factors in the Cattell–Horn–Carroll model of intelligence; McGrew, 2009). A recent study by Talipski et al. (2026) demonstrated precisely this. They developed a performance-based emotion recognition task using naturalistic (as opposed to posed) expressions and found that this test was correlated between .08 and .18 with cognitive empathy, but between .30 and .46 with general cognitive ability. This again provides evidence that emotion recognition is an ability which is indeed distinct, but cannot be considered entirely separately from general cognitive intelligence (Meyer et al., 2021; Walker et al., 2023, 2025; Wilhelm & Kyllonen, 2021).

This leads us to the (ironic) situation that, although the SEE-48 test failed as a measure of emotional intelligence, it appears to be a promising candidate for assessing analytical intelligence. It could be used as a non-verbal subtest of crystallised intelligence in a test that distinguishes between fluid and crystallised intelligence (Amthauer et al., 1999; Beauducel et al., 2001), or as a test of perceptual abilities in a test that distinguishes between verbal and perceptual skills (Haier et al., 2023; Johnson & Bouchard, 2005).

One last conclusion that we can draw from our findings is that, in future studies, performance-based emotion recognition tests should only be used alongside a vocabulary test. This will ensure that the conclusions relate to emotional intelligence rather than cognitive intelligence. For instance, Greenberg et al. (2023) conducted a large-scale study demonstrating that women outperform men on the RMET (d = 0.2). A similar advantage for women was seen in our study with the MRMET (d = 0.39) and STEU (d = 0.24), though to a lesser extent with the GERT (d = 0.16) and not with the SEE-48 (d = –0.14). Although there are no general differences in vocabulary knowledge between men and women, there are domain-specific differences corresponding to divergent interests in people versus things (Brysbaert, 2019). Of the 71 unique emotion labels used in the RMET for which prevalence scores are available, women are found to be more familiar with these words than men (d = 0.35). This suggests that the difference observed by Greenberg et al. (2023) could be due to gender differences in word knowledge rather than emotional intelligence.

Regardless of how our findings are interpreted, they confirm the observation that there is little correlation between performance-based tests of emotion recognition and self-reports of emotional skills (see also Heck et al., 2024; Murphy & Lilienfeld, 2019; Sunahara et al., 2022; Wendt et al., 2024; Xiao et al., 2025). Psychologists prefer performance-based tests over self-reports for good reason: the latter require insight into one’s performance level, a sufficiently large reference group and freedom from presentation bias. These requirements are rarely met, which is why objective, performance-based results are favoured in testing situations (Brown et al., 2023; Murphy & Lilienfeld, 2019; Neumann et al., 2015). However, this does not absolve researchers of the responsibility to ensure that their performance-based tests effectively measure what the researchers intend them to measure.

## Limitations

A first limitation of our study’s findings is the selection of tests used. Although we tried to include more than one test per construct, there are many more tests we could have included. We chose tests of performance-based emotion perception which overcame some of the limitations mentioned in the literature (Conway et al., 2024). For example, we used the MRMET rather than the original RMET, since this test was built with ground-truth in mind (the actors were asked to show the specific emotions), and incorporated objectively correct answers which could be judged accurately. The same was true for the SEE-48 and the GERT. Still, it is possible that emotions displayed by actors are not the same as truly felt emotions (Cong et al., 2025). Furthermore, we restricted ourselves to emotion perception with rather short and predominantly visual stimuli. Other tests may have higher correlations with social-emotional functioning. Also, emotion management and emotion use to make good decisions may be more important than emotion perception.

Secondly, the method used for our performance-based emotion perception tests differed from that used for most other variables. This may have contributed to the lack of correlation between the clusters, and the finding that all performance-based measures (including those that did not tap into emotion perception, such as the ART) clustered together. In this respect, our study invites further research into performance-based emotion tests that correlate more closely with real-life criteria, or the development of performance-based validation criteria (McLean et al., 2026). Alternatively, there may be other self-report criteria for social-emotional functioning for which performance-based emotion recognition tests could be useful predictors. Sunahara et al. (2022), for instance, reported a significant negative correlation between alexithymia ratings and RMET scores in a student population.

Thirdly, even after modification, our SEM showed only marginal fit. This leaves open the possibility that a different structure will be found if the authors manage to find a better set of tests (hopefully partially based on our findings). While we are fairly confident in our conclusions, as we observed the same structure in the intercorrelation matrix and the EGA analysis as in the SEM, we recognise that others may be more sceptical.

Fourth, our study was limited to a neuro-typical population. More information may be gathered from groups that have reduced emotion perception in the presence of non-reduced crystallised intelligence (see Oakley et al., 2016). Possible groups are people above the age of 70, people with autism-spectrum disorder, or people affected by traumatic brain injury. Alternatively, we can investigate whether emotion recognition tests predict performance in professions where this skill is of importance (McLean et al., 2026). Importantly, in these studies it will be critical to include good tests of (crystallised) intelligence to make sure that any effect is specific to emotion processing.

Fifth, our participants were tested online and came from Prolific’s participant pool. This was the only way we could test a large sample of non-students within the time frame we had. Ideally, the study would be repeated with a more diverse sample of participants tested face to face.

Finally, our findings with self-assessments are valid only for the study context used. Participants did not expect outcomes to depend on the answers given. This is different from selection situations, where participants expect certain profiles to be at an advantage and are likely to adjust their self-evaluations according to these expectations (Dunlop et al., 2020).

## Additional File

The additional file for this article can be found as follows:

10.5334/pb.1443.s1 Supplementary Materials. The Supplementary Materials file contains more detailed information about the tests that were used and step-by-step code for the analyses that were run in this article.

## Data Availability

Data and code for all R analyses reported in this manuscript is available at https://osf.io/kcqe4/.

## Footnotes

A similar number comes from an analysis with G*Power. To detect correlations of r = 0.20 with a (one-tailed) significance level alpha = 0.050 and with a power beta = 0.900, a formal power analysis using an exact correlation test assuming a bivariate normal distribution suggests that the required sample size equals N = 211. We thank an anonymous reviewer for pointing this out to us.
