# Supplementary material for: On the Construct Validity of Performance-Based Emotion Recognition Tests: Correlations with Social-Emotional Functioning and Cognitive Skills
Source: Psychol Belg. 2026 May 25;66(1):47–64. doi: 10.5334/pb.1443 (PMC13220741; doi:10.5334/pb.1443)

Supplementary materials Hendel & Brysbaert

Tests and analysis

This text gives more information about the tests we used and the analyses we did. The analyses can be run with the R file or the Markdown file.

The raw data are stored in the file "Final Data.xlsx". It contains the cleaned data of the Prolific study. Cleaning involved deleting participants who tried out the study, did not complete the whole battery, or gave indications of careless responding. It left 227 participants (rows), responding to 630 items (columns + 20 columns with information about the participants).

```
Final_Data <- read_excel("Final Data.xlsx")
```

The following questionnaires/tests were used.

1. **Reading absorption**

   This test is also known as the MODTAS (Jamieson, 2005). It contains 34 questions, which largely measure a single factor (see scree plot in Figure 1 of Jamieson, 2005), even though the author preferred an analysis with 5 intercorrelated primary factors and a single higher order factor. Likert scale going from 0 to 4. We simply took the mean score (equivalent to the sum score).

```
MODTAS <- Final_Data[,21:54]
Tests_participants$Reading_absorption <- rowMeans(MODTAS)
```

2. **Preference for and competence in jobs involving people or things**

   Based on the Personal Globe Inventory – Short (PGI-Short; Tracey, 2010). It contains 40 questions, which are given twice: once for liking and once for feeling of competence. Several keys are given in Appendix B. The one used here is “People/Things”. It involves the difference score between 16 questions about people jobs and 16 questions about things jobs. Likert scale from 1 to 7. We again took the mean scores.

```
Pgi_short <- Final_Data[,55:134]
Tests_participants$Liking_jobs_people <- rowMeans(Pgi_short_liking_people) -
rowMeans(Pgi_short_liking_things)
Tests_participants$Competence_jobs_people <-
rowMeans(Pgi_short_competence_people) -
rowMeans(Pgi_short_competence_things)
```

3. **Social curiosity**

   Based on Renner (2010). The first five questions are about over social curiosity; the last 5 questions are about covert social curiosity. Likert scale from 1 to 4. Mean scores.

```
SCS <- Final_Data[,135:144]
Tests_participants$Social_curiosity <- rowMeans(SCS[,1:5])
```

```
Tests_participants$Covert_curiosity <- rowMeans(SCS[,6:10])
```

4. **Loneliness**

UCLA Loneliness Scale (Russell, 1996). Twenty questions. Likert scale from 1 to 4. Mean scores.

```
UCLA <- Final_Data[,145:164]
# Change reverse coded items
UCLA[,c(1,5,6,9,10,15,16,19,20)] <- 5 - UCLA[,c(1,5,6,9,10,15,16,19,20)]
Tests_participants$Loneliness <- rowMeans(UCLA)
```

5. **Social efficacy**

Based on Irwing et al. (2024). Contains five questions. Likert scale from 1 to 5. Mean scores.

```
Efficacy <- Final_Data[,165:169]
Tests_participants$Social_efficacy <- rowMeans(Efficacy)
```

6. **Social confidence**

Based on Irwing et al. (2024). Contains six questions. Likert scale from 1 to 7. Mean scores.

```
Confidence <- Final_Data[,170:175]
Tests_participants$Social_confidence <- rowMeans(Confidence)
```

7. **SEE-48**

Performance-based test of emotion recognition (Franca et al., 2023). Participants must recognize six primary emotions in actors' eyes. 48 trials. Correct/incorrect. Mean score.

```
SEE_48 <- Final_Data[,176:223]
Tests_participants$SEE_48 <- rowMeans(SEE_48)
```

8. **STEU**

Performance-based test of emotion understanding based on McCann & Roberts (2008). Short descriptions of situations. Participants must indicate which feeling the protagonist is likely to experience. Originally contained 42 items. Based on item analysis of various datasets, the 25 best items were retained (e.g., many items that make a distinction between angry and frustrated do not score well). The items are listed in Appendix A (Table 1). Correct/incorrect. Mean score.

```
STEU <- Final_Data[,224:248]
Tests_participants$STEU <- rowMeans(STEU)
```

9. **MRMET**

New version of RMET, published by Kim et al. 2024. Participants must recognize emotions in eyes. Better psychometric characteristics than RMET, no biased items, less response alternatives. 37 items. Correct/incorrect. Mean score.

```
MRMET <- Final_Data[,249:285]
Tests_participants$MRMET <- rowMeans(MRMET)
```

10. **GERT**

Geneva Emotion Recognition Test (Schlegel & Scherer, 2016). Contains 42 short videos of actors performing emotions (with sound, but no meaningful language). Cannot be shared because the test is copyright protected. Correct/incorrect. Mean score.

```
GERT <- Final_Data[,286:327]
Tests_participants$GERT <- rowMeans(GERT)
```

11. **Preference for jobs with people or things**

Newly created test in which participants must indicate their preference for jobs, one of which involves working with people, the other without. 30 items; alternative selected. The items are shown in Appendix A (Table 2). Initially scored for preference

for things; reversed to preference for people. Percentage of people-alternatives selected.

```
Occupations <- Final_Data[,328:357]
Tests_participants$Occupations_people <- 1 - rowMeans(Occupations)
```

12. **Self-reported empathy**

Questionnaire of Cognitive and Affective Empathy (Reniers et al., 2010). Contains 31 items, measuring 5 factors: perspective taking, online simulation, emotion contagion, proximal responsivity, peripheral responsivity. Likert scale from 1 to 4. Mean scores.

```
QCAE <- Final_Data[,358:404]
perspective_taking <- QCAE[,c(5,9,13,17,18,19,23,26,33,44)]
emotion_contagion <- QCAE[,c(2, 3, 15, 24)]
online_simulation <- QCAE[,c(7,21,27,28,31,37,40,43,45)]
online_simulation$QCAE_21 <- 5-online_simulation$QCAE_21
peripheral_responsivity <- QCAE[,c(32,34,39,47)]
peripheral_responsivity <- 5-peripheral_responsivity
peripheral_responsivity$QCAE_34 <- 5-peripheral_responsivity$QCAE_34
proximal_responsivity <- QCAE[,c(11,20,30,41)]
```

13. **Metacognition emotion perception**

Newly created test that asks people how difficult it is for them to recognize emotions from the eyes of others. 16 questions (Appendix A, Table 3). Likert scale from 1 to 4, all reverse scored. Presented randomly interspersed with items of the QCAE, because the latter contains very few reversely scored items. Also meant as an attention check to detect careless responding.

```
metacognition_emotion_perception <-
QCAE[,c(1,4,6,8,10,12,14,16,22,25,29,35,36,38,42,46)]
metacognition_emotion_perception <- 5-metacognition_emotion_perception
```

14. **Life satisfaction**

Satisfaction With Life Scale (Diener, 1985). Five questions, Likert scale from 1 to 7. Mean scores.

```
Life_satisfaction <- Final_Data[,405:409]
Tests_participants$Life_satisfaction <- rowMeans(Life_satisfaction)
```

15. Lubben relations

This scale measures the number and closeness of the relationships participants have with family and friends (Lubben et al., 2006). Participants are asked to respond to three questions about family and three questions pertaining to friendships on a 5-point scale ranging from “0 = none” to “5 = nine or more”. Mean scores.

```
Lubben_relations <- Final_Data[,410:415]
Tests_participants$Lubben_relations <- rowMeans(Lubben_relations)
```

16. Rejection sensitivity

Adult Rejection Sensitivity Questionnaire (Berenson et al., 2009). Contains 18 questions, largely assessing a single factor. Likert scale from 1 to 6. Mean scores.

```
Rejection_sensitivity <- Final_Data[,416:433]
Rejection_sensitivity[,10:18] <- 7 - Rejection_sensitivity[,10:18]
Tests_participants$Rejection_sensitivity <- rowMeans(Rejection_sensitivity)
```

17. Reading motivation

Predictors of Leisure Reading (PoLR) scale (Martin-Chang et al., 2021). Three factors were included: reading motivators, demotivators and attitudes towards reading for leisure. Contains 16 statements (Appendix A, Table 4). Likert scale from 1 to 7. Mean scores.

```
Leisure_reading <- Final_Data[,434:449]
Tests_participants$Reading_motivation <- rowMeans(Leisure_reading[,1:5])
Tests_participants$Reading_demotivation <- rowMeans(Leisure_reading[,6:10])
Tests_participants$Reading_attitude <- rowMeans(Leisure_reading[,11:16])
```

18. Reading habits

Based on Kuijpers et al. (2020). Contains 21 different reading genres. Participants must indicate from 0 to 6 how often they read each genre. Divided into fiction and non-fiction; social media and email were excluded, as it is not clear to which category they belong. Mean scores.

```
Reading_habits <- Final_Data[,450:470]
Tests_participants$Reading_fiction <-
rowMeans(Reading_habits[,c(1,2,4,5,7,9,10,11,12,13,16,20,21)])
Tests_participants$Reading_non.fiction <-
rowMeans(Reading_habits[,c(3,6,8,14,15,19)])
```

19. Author recognition test

Taken from Vermeiren et al. (2023). Has 90 items: 60 author names and 30 foil names. Participants must indicate the authors they know. Score is percentage authors correct minus percentage foils indicated as author.

```
ART <- Final_Data[,471:560]
Tests_participants$ART <- rowMeans(authors) - (1 - rowMeans(nonaut))
```

20. Vocabulary test

Newly formed test, based on Vermeiren et al. (2023) (Appendix A, Table 5). We combined items of StuVoc1 and StuVoc3 in case we wanted to replicate the study with English L2 speakers. Has 50 multiple-choice items. Percentage correct. The scores are slightly skewed because the test was a bit too easy for the participants who took part.

```
Stuvoc <- Final_Data[,561:610]
Tests_participants$Stuvoc <- rowMeans(Stuvoc)
```

21. General knowledge test

Taken from Vermeiren et al. (2023). Has 40 multiple-choice items. Percentage correct.

```
GK <- Final_Data[,611:650]
Tests_participants$GK <- rowMeans(GK)
```

Descriptive statistics

library(psych)
describe(Tests_participants)

The data are close to normally distributed.

	vars	n	mean	sd	median	trimmed	mad	min	max	range	skew	kurtosis	se
Reading_absorption	3	227	1.56	0.69	1.56	1.55	0.70	0.06	3.59	3.53	0.21	-0.46	0.05
Liking_jobs_people	4	227	0.74	1.12	0.69	0.74	1.11	-2.94	3.56	6.50	-0.06	0.20	0.07
Competence_jobs_people	5	227	0.91	1.02	0.94	0.92	1.02	-2.19	3.69	5.88	-0.14	0.16	0.07
Social_curiosity	6	227	3.00	0.53	3.00	3.01	0.30	1.20	4.00	2.80	-0.38	0.62	0.04
Covert_curiosity	7	227	2.58	0.67	2.60	2.60	0.59	1.00	4.00	3.00	-0.16	-0.20	0.04
Loneliness	8	227	2.34	0.64	2.35	2.34	0.67	1.00	3.70	2.70	-0.04	-0.83	0.04
Social_efficacy	9	227	3.92	0.70	4.00	3.95	0.59	1.60	5.00	3.40	-0.38	-0.29	0.05
Social_confidence	10	227	3.84	1.26	3.83	3.85	1.24	1.00	7.00	6.00	0.00	-0.61	0.08
SEE_48	11	227	0.63	0.11	0.65	0.64	0.09	0.27	0.90	0.62	-0.29	-0.15	0.01
STEU	12	227	0.70	0.12	0.72	0.70	0.12	0.28	0.96	0.68	-0.41	0.02	0.01
MRMET	13	227	0.69	0.11	0.70	0.70	0.12	0.41	0.92	0.51	-0.42	-0.44	0.01
GERT	14	227	0.56	0.13	0.57	0.57	0.14	0.26	0.81	0.55	-0.13	-0.69	0.01
Occupations_people	15	227	-0.50	0.20	-0.50	-0.50	0.25	-0.93	-0.07	0.87	0.04	-0.88	0.01
QCAE_perspective_taking	16	227	2.84	0.49	2.90	2.87	0.44	1.20	4.00	2.80	-0.61	0.94	0.03
QCAE_online_simulation	17	227	2.98	0.52	3.00	3.00	0.49	1.00	4.00	3.00	-0.39	0.56	0.03
QCAE_emotion_contagion	18	227	2.81	0.64	2.75	2.83	0.74	1.00	4.00	3.00	-0.37	-0.09	0.04
QCAE_proximal_responsivity	19	227	2.89	0.61	3.00	2.91	0.74	1.00	4.00	3.00	-0.39	-0.04	0.04
QCAE_peripheral_responsivity	20	227	2.82	0.61	2.75	2.83	0.74	1.00	4.00	3.00	-0.19	-0.15	0.04
metacognition_emotion_perception	21	227	3.14	0.55	3.06	3.16	0.56	1.25	4.00	2.75	-0.19	-0.33	0.04
Life_satisfaction	22	227	4.05	1.49	4.20	4.10	1.78	1.00	7.00	6.00	-0.24	-0.80	0.10
Lubben_relations	23	227	3.44	0.91	3.50	3.44	0.99	1.17	6.00	4.83	-0.01	-0.46	0.06
Rejection_concern	24	227	3.53	0.98	3.44	3.53	0.99	1.11	6.00	4.89	0.08	-0.34	0.06
Rejection_expectancy	25	227	2.50	0.70	2.44	2.48	0.66	1.11	4.89	3.78	0.34	-0.14	0.05

Reading_motivation	26	227	5.33	1.30	5.60	5.49	1.19	1.00	7.00	6.00	-1.27	1.71	0.09
Reading_demotivation	27	227	2.61	1.54	2.20	2.43	1.78	1.00	7.00	6.00	0.82	-0.29	0.10
Reading_attitude	28	227	5.32	0.89	5.33	5.35	0.99	1.83	7.00	5.17	-0.64	1.10	0.06
ART	29	227	0.35	0.21	0.32	0.34	0.20	-0.08	1.00	1.08	0.63	0.13	0.01
Reading_fiction	30	227	1.77	1.11	1.69	1.72	1.14	0.00	4.62	4.62	0.35	-0.69	0.07
Reading_non.fiction	31	227	2.25	1.28	2.17	2.19	1.24	0.00	6.00	6.00	0.42	-0.20	0.08
Stuvoc	32	227	0.85	0.13	0.88	0.87	0.12	0.34	1.00	0.66	-1.36	1.57	0.01
GK	33	227	0.73	0.13	0.75	0.73	0.15	0.38	1.00	0.62	-0.21	-0.59	0.01

Correlation matrix

Nothing is more informative than a correlation matrix. Even though the variables are normally distributed, we calculated both Pearson correlations (above the diagonal) and Spearman correlations (below the diagonal). We ordered the correlations according to hclust (based on scaled data), which gives a first indication of the structure in the variables. For this the data were standardized (has no effect on the correlations, but is needed for hclust).

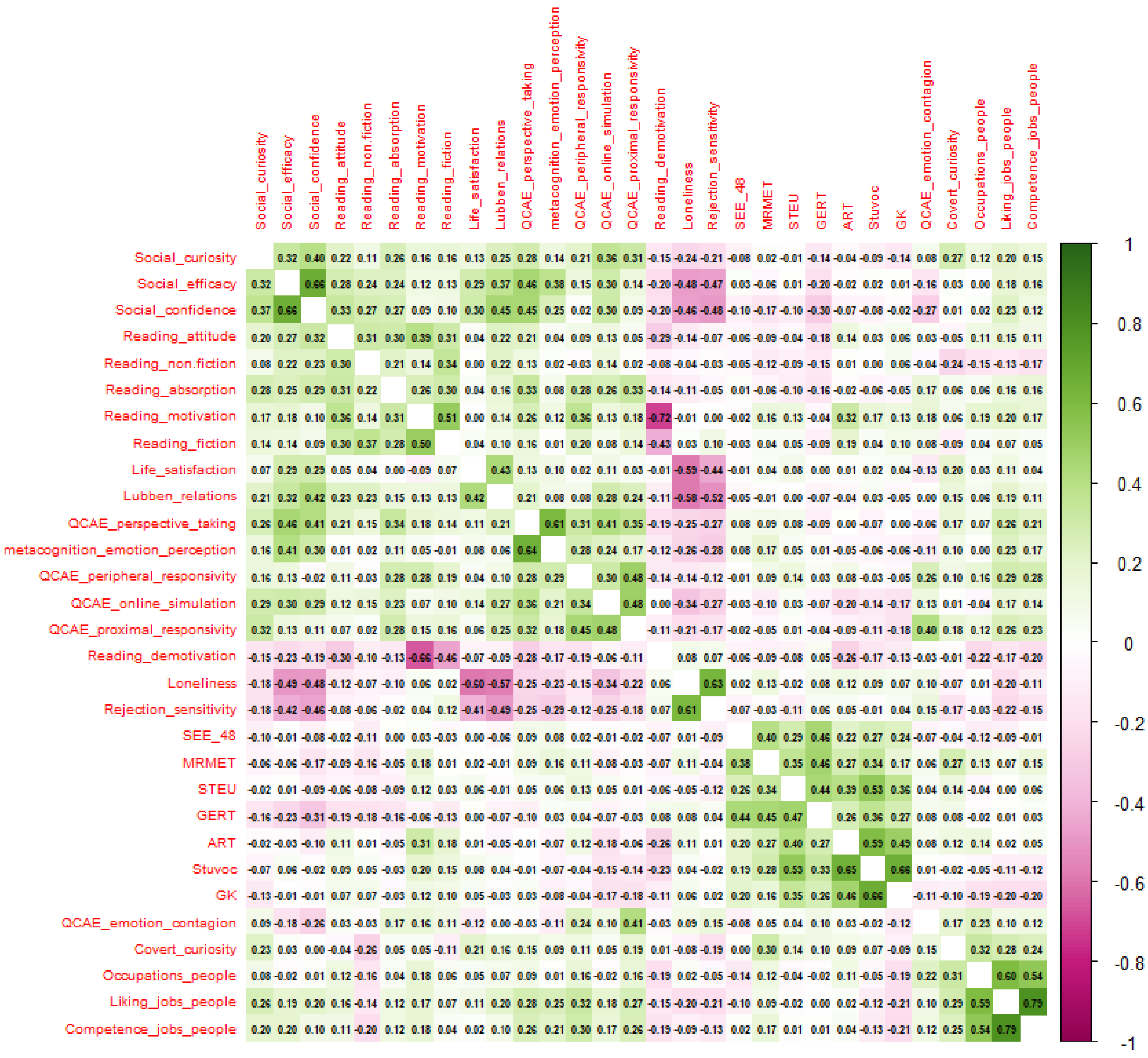

Figure 1: Correlations between the tests.

There are three main clusters: One on the top left, including socio-emotional functioning, reading, and self-reported empathy. A second one is in the middle, including the performance-based tests of emotion recognition/understanding and the tests of crystallized intelligence, and a third one in the right bottom corner, involving a preference for jobs with people. Further important is the almost complete absence of positive correlations between cluster 1 and 2. The largest positive correlation is between reading motivation and scores on the author recognition test (ART). There are even negative correlations between scores on the GERT test and social efficacy/confidence. Also, the correlations between cluster 2 and 3 are largely absent, the only exception being MRMET, which seems to have some positive correlations, in particular with covert social curiosity.

Exploratory graph analysis complete dataset

A way to get a better feeling for the structure is to look at an exploratory graph analysis. We use the package EGAnet, as this seems to have been well thought through (with good default values; Christensen et al., 2020).

```
emo_ega <- EGA(Tests_participants[,3:ncol(Tests_participants)])
emo_ega
```

This analysis suggests 5 clusters. The big cluster of Figure1 is divided into three smaller clusters: self-reported empathy, reading, and social-emotional functioning. The cluster of performance-based emotion recognition and tests of crystallized intelligence is almost completely separated from the rest. The only exceptions are the positive links between MRMET and covert social curiosity, between the author recognition test and reading motivation, and the negative link between GERT and social confidence.

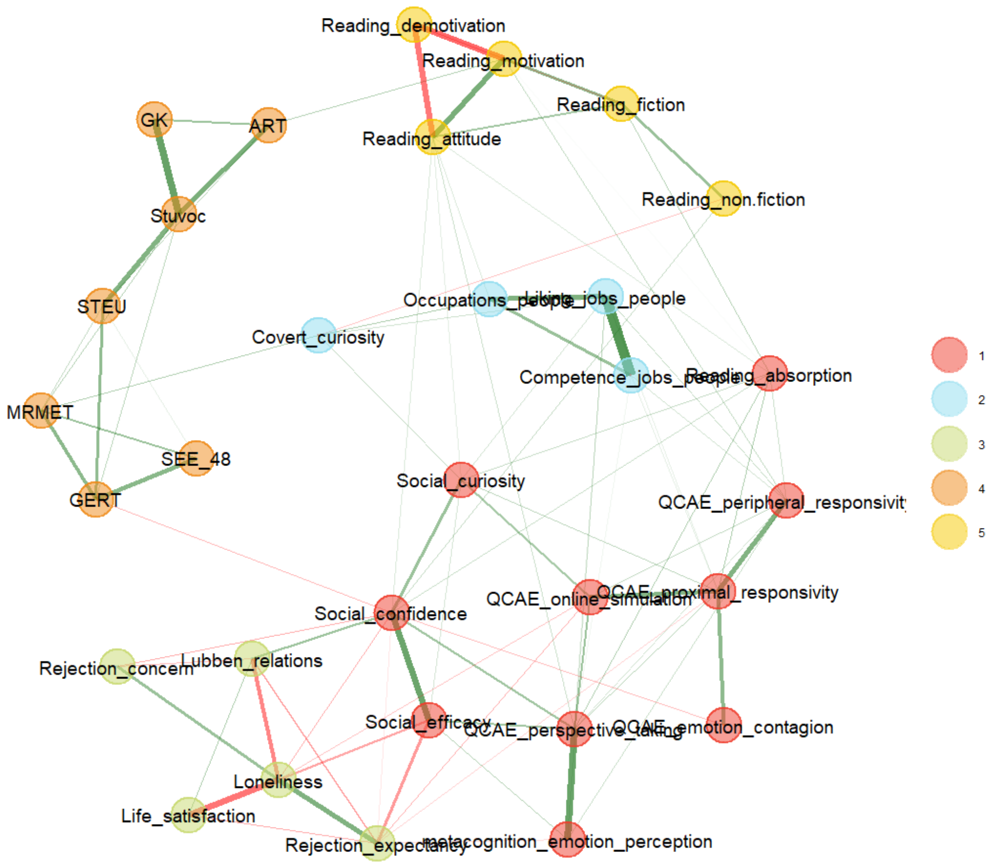

Figure 2: Outcome of an exploratory graph analysis when all tests are included.

Boot-strapping indicates that the above clustering agrees with the most frequent clustering and shows which tests are most robust for each cluster.

```
emo_boot <- bootEGA(
  data = Tests_participants[,2:ncol(Tests_participants)],
  seed = 12112025 # set seed for reproducibility
)
```

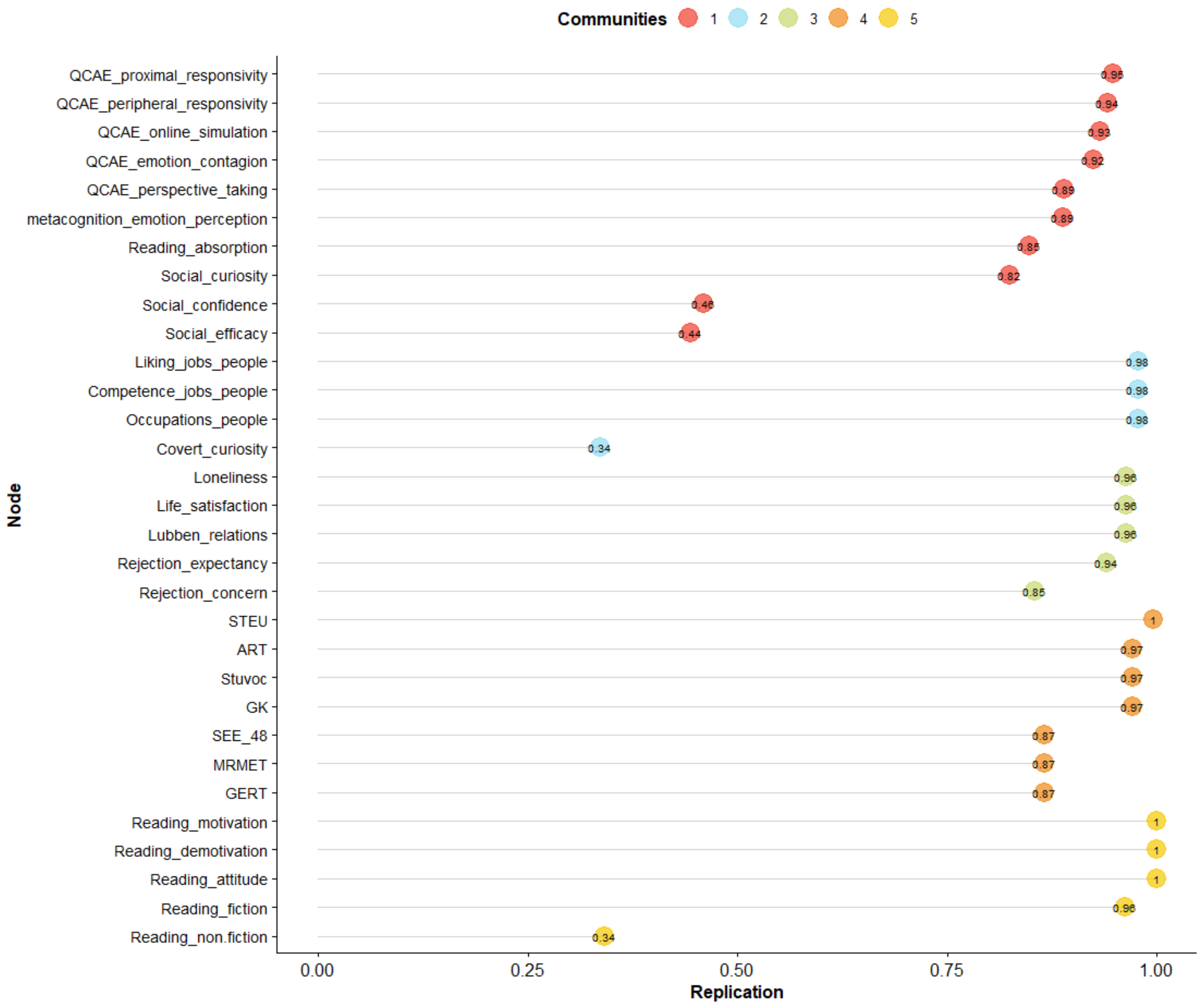

Figure 3: Number of times each test remains in its cluster when random samples of the data are taken (bootstrapping).

The end structure of bootstrapping was the same as the structure found in the initial analysis.

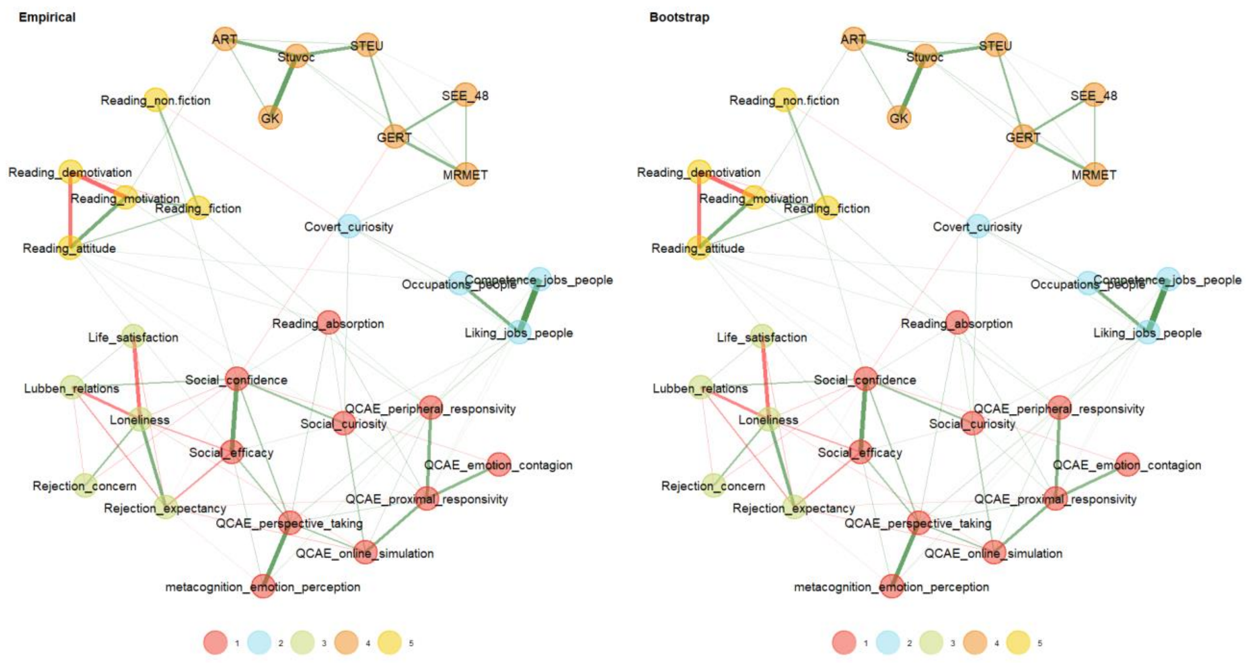

Figure 4: Network organization after bootstrapping is the same as the organization of the initial analysis restricted to the observed data.

We can have a look at the clusters in which the various tests end up in the 500 bootstraps:

```
emo.dimstab <- dimensionStability(emo_boot)
emo.dimstab$item.stability$item.stability$all.dimensions
```

	1	2	3	4	5	6	7	8	9
Reading_absorption	0.848	0.004	0.008	0.002	0.058	0.054	0.022	0.002	0.000
Liking_jobs_people	0.018	0.978	0.000	0.000	0.000	0.002	0.002	0.000	0.000
Competence_jobs_people	0.018	0.978	0.000	0.000	0.000	0.002	0.002	0.000	0.000
Social_curiosity	0.824	0.022	0.048	0.000	0.010	0.062	0.026	0.008	0.000
Covert_curiosity	0.310	0.336	0.038	0.050	0.066	0.132	0.050	0.012	0.000
Loneliness	0.022	0.000	0.964	0.000	0.000	0.012	0.002	0.000	0.000
Social_efficacy	0.444	0.000	0.476	0.000	0.000	0.058	0.022	0.000	0.000
Social_confidence	0.460	0.000	0.456	0.000	0.000	0.062	0.022	0.000	0.000
SEE_48	0.000	0.000	0.000	0.866	0.000	0.120	0.014	0.000	0.000
STEU	0.000	0.000	0.000	0.996	0.000	0.004	0.000	0.000	0.000
MRMET	0.000	0.000	0.000	0.866	0.000	0.120	0.014	0.000	0.000
GERT	0.000	0.000	0.000	0.866	0.000	0.120	0.014	0.000	0.000
Occupations_people	0.018	0.978	0.000	0.000	0.000	0.002	0.002	0.000	0.000
QCAE_perspective_taking	0.890	0.000	0.024	0.000	0.000	0.062	0.022	0.000	0.002
QCAE_online_simulation	0.932	0.000	0.014	0.000	0.000	0.044	0.010	0.000	0.000
QCAE_emotion_contagion	0.924	0.002	0.016	0.000	0.000	0.040	0.018	0.000	0.000
QCAE_proximal_responsivity	0.948	0.000	0.000	0.000	0.000	0.036	0.016	0.000	0.000
QCAE_peripheral_responsivity	0.942	0.006	0.000	0.000	0.002	0.036	0.014	0.000	0.000
metacognition_emotion_perception	0.888	0.000	0.026	0.000	0.000	0.062	0.022	0.000	0.002
Life_satisfaction	0.022	0.000	0.964	0.000	0.000	0.012	0.002	0.000	0.000
Lubben_relations	0.022	0.000	0.964	0.000	0.000	0.012	0.002	0.000	0.000
Rejection_concern	0.102	0.000	0.854	0.000	0.000	0.026	0.018	0.000	0.000
Rejection_expectancy	0.046	0.000	0.940	0.000	0.000	0.012	0.002	0.000	0.000
Reading_motivation	0.000	0.000	0.000	0.000	1.000	0.000	0.000	0.000	0.000
Reading_demotivation	0.000	0.000	0.000	0.000	1.000	0.000	0.000	0.000	0.000
Reading_attitude	0.000	0.000	0.000	0.000	1.000	0.000	0.000	0.000	0.000
ART	0.000	0.000	0.000	0.972	0.000	0.028	0.000	0.000	0.000
Reading_fiction	0.014	0.002	0.000	0.000	0.962	0.012	0.008	0.002	0.000
Reading_non.fiction	0.292	0.118	0.044	0.014	0.342	0.128	0.050	0.008	0.000
Stuvoc	0.000	0.000	0.000	0.972	0.000	0.028	0.000	0.000	0.000
GK	0.000	0.000	0.000	0.972	0.000	0.028	0.000	0.000	0.000

To have an idea of the correlations between the clusters, we can run a hierarchical EGA analysis:

```
emo.hier <- hierEGA(Tests_participants[,3:ncol(Tests_participants)])
```

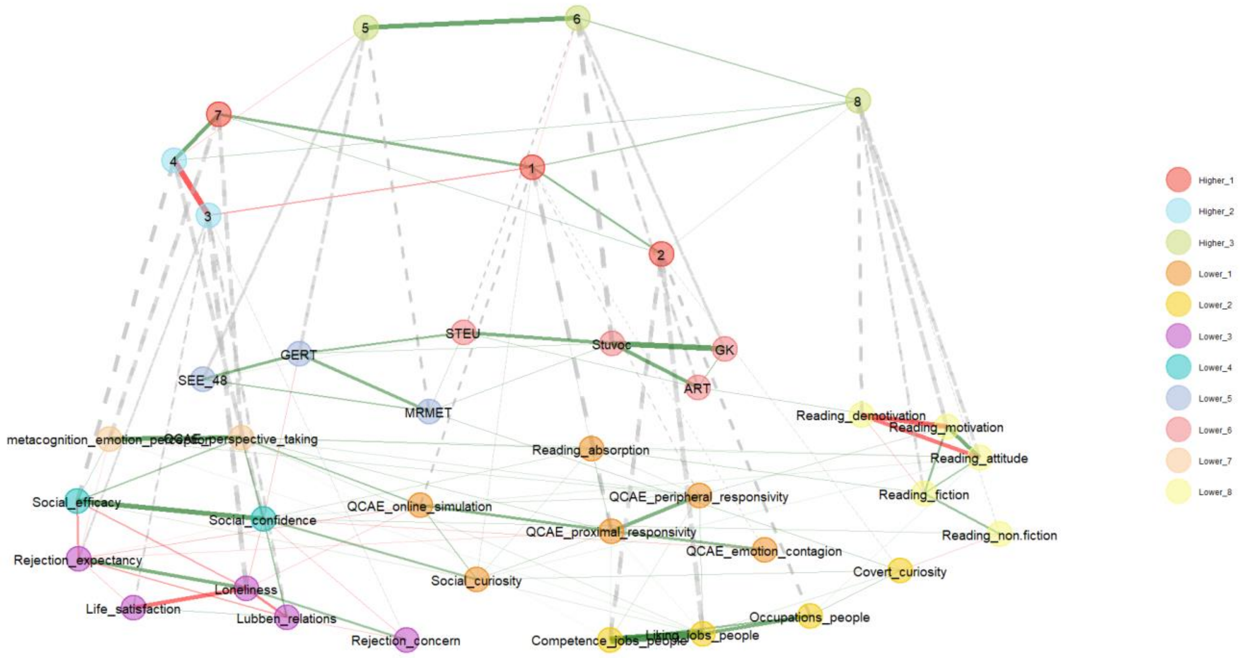

Figure 5: Hierarchical EGA analysis of the tests.

The most interesting finding here is that the cluster of performance-based tests breaks down into a cluster with emotion recognition tests (SEE-48, GERT, MRMET) and a cluster with crystallised intelligence (Stuvoc, GK, ART), to which STEU also belongs (this test actually is part of both factors when analysed with SEM).

Exploratory graph analysis with non-redundant tests

A potential issue with analyses with all tests is that they may be biased by redundant tests. These are test pairs that have a higher correlation than expected on the basis of the network. Christensen et al. (2023) describe an algorithm to detect local dependencies. The code to use in the EGAnet package is:

```
part_uva <- UVA(
  data = Tests_participants[,3:ncol(Tests_participants)])
part_uva
```

This tells us that the following are redundant items pairs (they can also be seen in the correlation matrix, as the item pairs with very high correlations):

Variable pairs with wTO > 0.30 (large-to-very large redundancy)

node_i	node_j	wto
Liking_jobs_people	Competence_jobs_people	0.464
Reading_motivation	Reading_demotivation	0.427
Stuvoc	GK	0.354
QCAE_perspective_taking	metacognition_emotion_perception	0.323

Variable pairs with wTO > 0.25 (moderate-to-large redundancy)

node_i	node_j	wto
Loneliness	Life_satisfaction	0.282
Social_efficacy	Social_confidence	0.265
Liking_jobs_people	Occupations_people	0.257
ART	Stuvoc	0.254

From each pair of highly correlated nodes, the program selects the item that contributes most to the network. Then the exploratory graph analysis can be repeated. Notice that Stuvoc, GK, and ART are projected to have too high intercorrelations. This hollows out the factor of crystallised intelligence. The same is true for the factor of liking jobs with people, where the three tests have too high intercorrelations.

```
emo_ega <- EGA(part_uva$reduced_data)
```

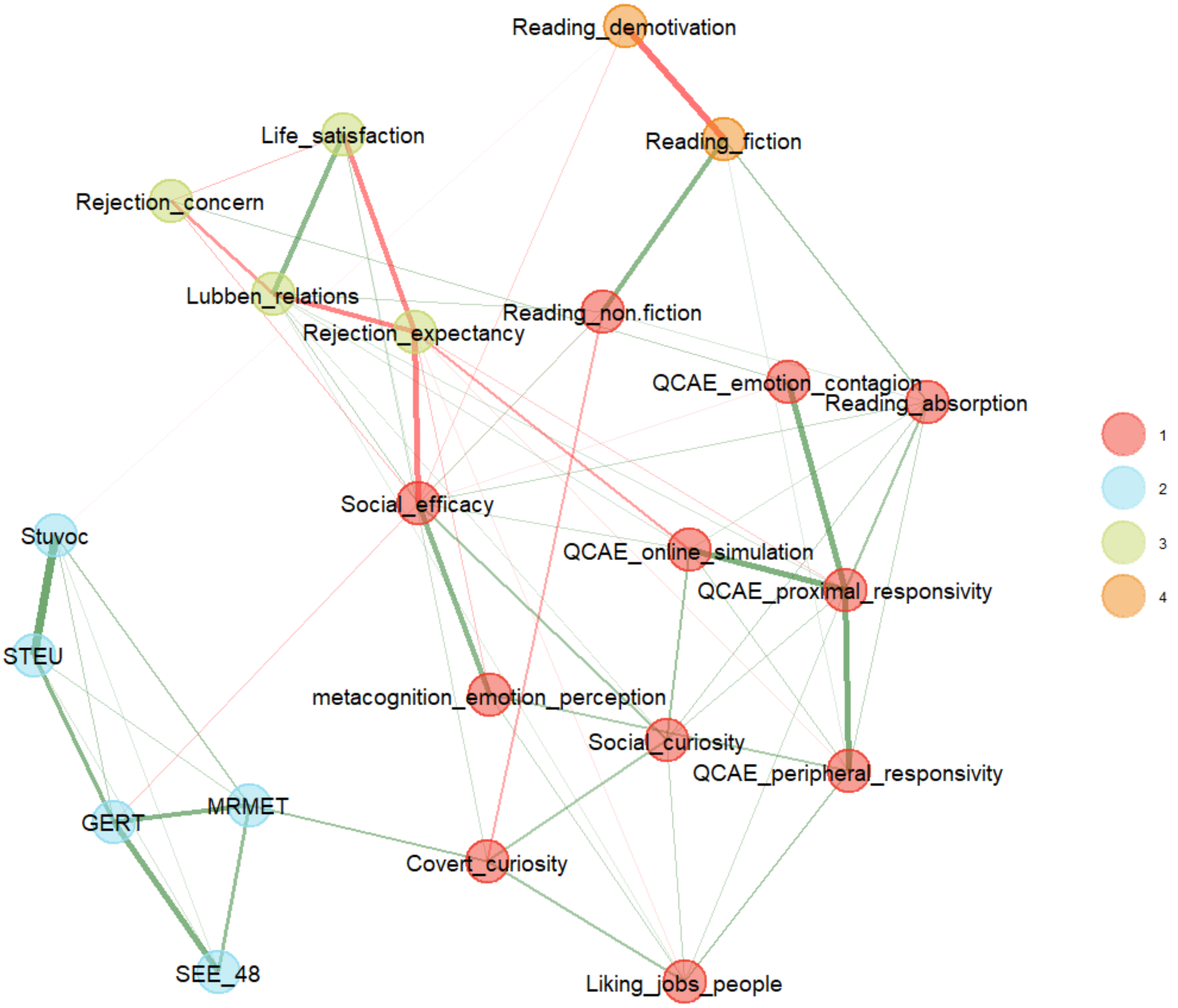

Figure 6: Outcome of an exploratory graph analysis when redundant items are dropped.

Not much changes, except that the clusters “liking jobs with people” and “self-ratings” merge.

Bootstrapping tells us which tests are stable estimates of the clusters and which possibly belong to more than one cluster.

```
emo_boot <- bootEGA(
  data = part_uva$reduced_data,
  seed = 12112025 # set seed for reproducibility
)
```

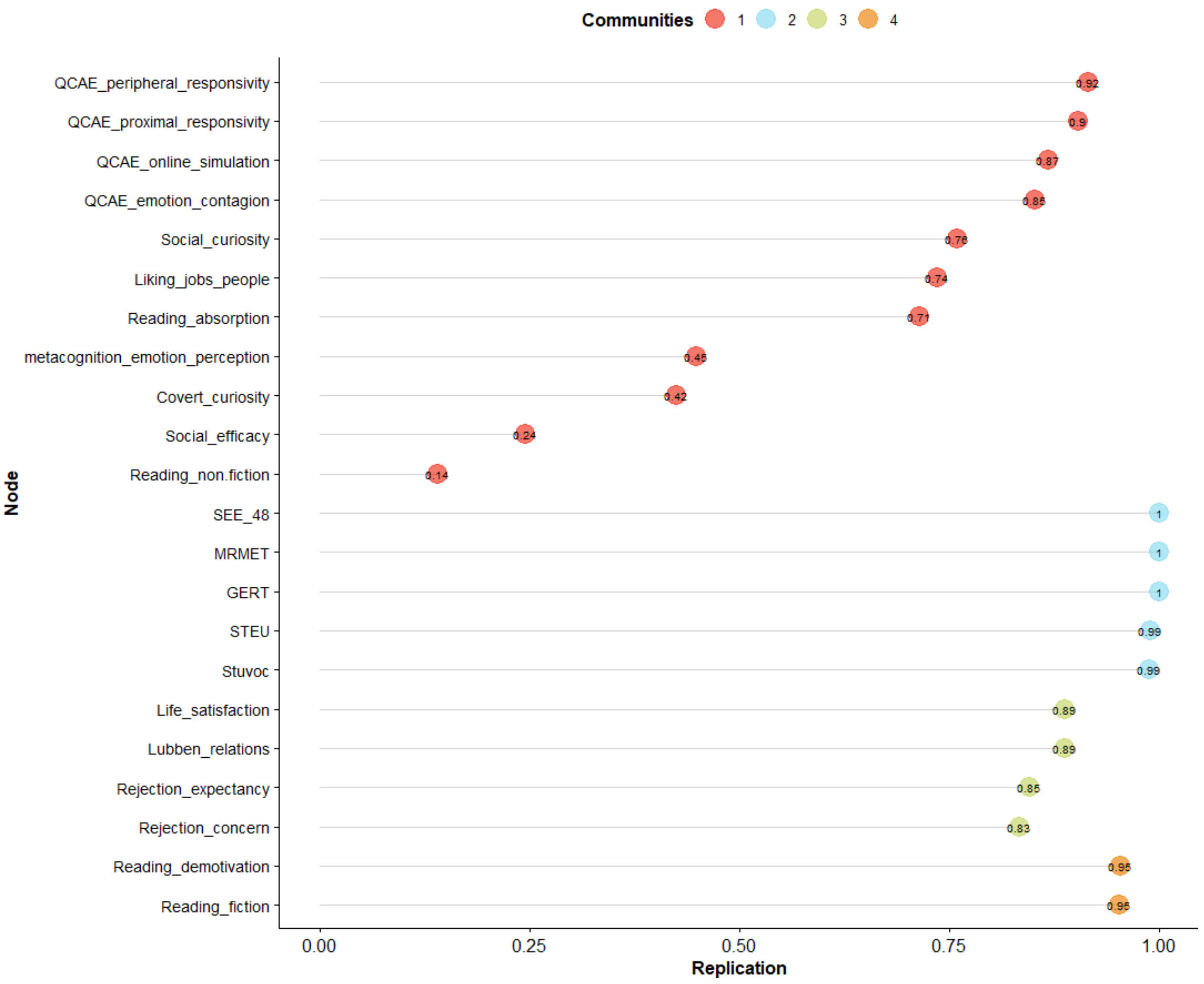


```
emo_compare <- compare.EGA.plots(
```

```
  emo_ega, emo_boot,
  labels = c("Empirical", "Bootstrap")
)
```

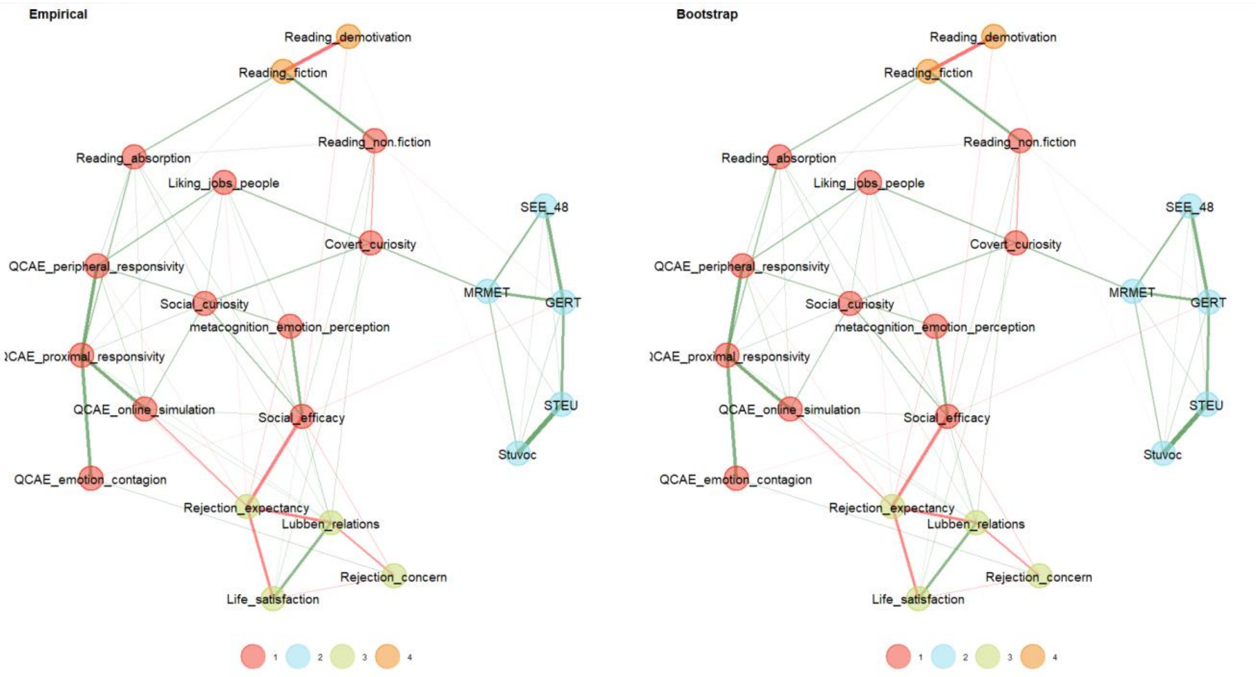

```
emo.dimstab <- dimensionStability(emo_boot)
emo.dimstab$item.stability$item.stability$all.dimensions
```

```
                                      1     2     3     4     5     6     7
Reading_absorption                0.714 0.000 0.016 0.184 0.072 0.010 0.000
Liking_jobs_people                0.736 0.000 0.052 0.048 0.152 0.008 0.000
Social_curiosity                  0.760 0.000 0.070 0.036 0.128 0.006 0.000
Covert_curiosity                  0.424 0.090 0.066 0.164 0.210 0.012 0.000
Social_efficacy                   0.244 0.000 0.578 0.014 0.146 0.016 0.000
SEE_48                            0.000 1.000 0.000 0.000 0.000 0.000 0.000
STEU                              0.000 0.990 0.000 0.000 0.000 0.008 0.002
MRMET                             0.000 1.000 0.000 0.000 0.000 0.000 0.000
GERT                              0.000 1.000 0.000 0.000 0.000 0.000 0.000
QCAE_online_simulation            0.868 0.000 0.030 0.010 0.088 0.004 0.000
QCAE_emotion_contagion            0.852 0.000 0.044 0.010 0.088 0.004 0.000
QCAE_proximal_responsivity        0.904 0.000 0.006 0.010 0.076 0.004 0.000
QCAE_peripheral_responsivity      0.916 0.000 0.004 0.012 0.066 0.002 0.000
metacognition_emotion_perception  0.448 0.000 0.366 0.018 0.156 0.012 0.000
Life_satisfaction                 0.020 0.000 0.888 0.002 0.084 0.006 0.000
Lubben_relations                  0.020 0.000 0.888 0.002 0.084 0.006 0.000
Rejection_concern                 0.048 0.000 0.834 0.006 0.100 0.006 0.000
Rejection_expectancy              0.040 0.000 0.846 0.004 0.102 0.008 0.000
Reading_demotivation              0.018 0.000 0.014 0.954 0.014 0.000 0.000
Reading_fiction                   0.018 0.000 0.014 0.952 0.016 0.000 0.000
Reading_non.fiction               0.140 0.000 0.044 0.670 0.114 0.016 0.000
Stuvoc                            0.000 0.988 0.000 0.000 0.000 0.008 0.002
```

To look at the intercorrelations between the clusters, we can again run a hierarchical graph analysis. This examines whether the first-order clusters can be grouped in second-order clusters (similar to a hierarchical cluster analysis).

hierEGA(part_uva$reduced_data)

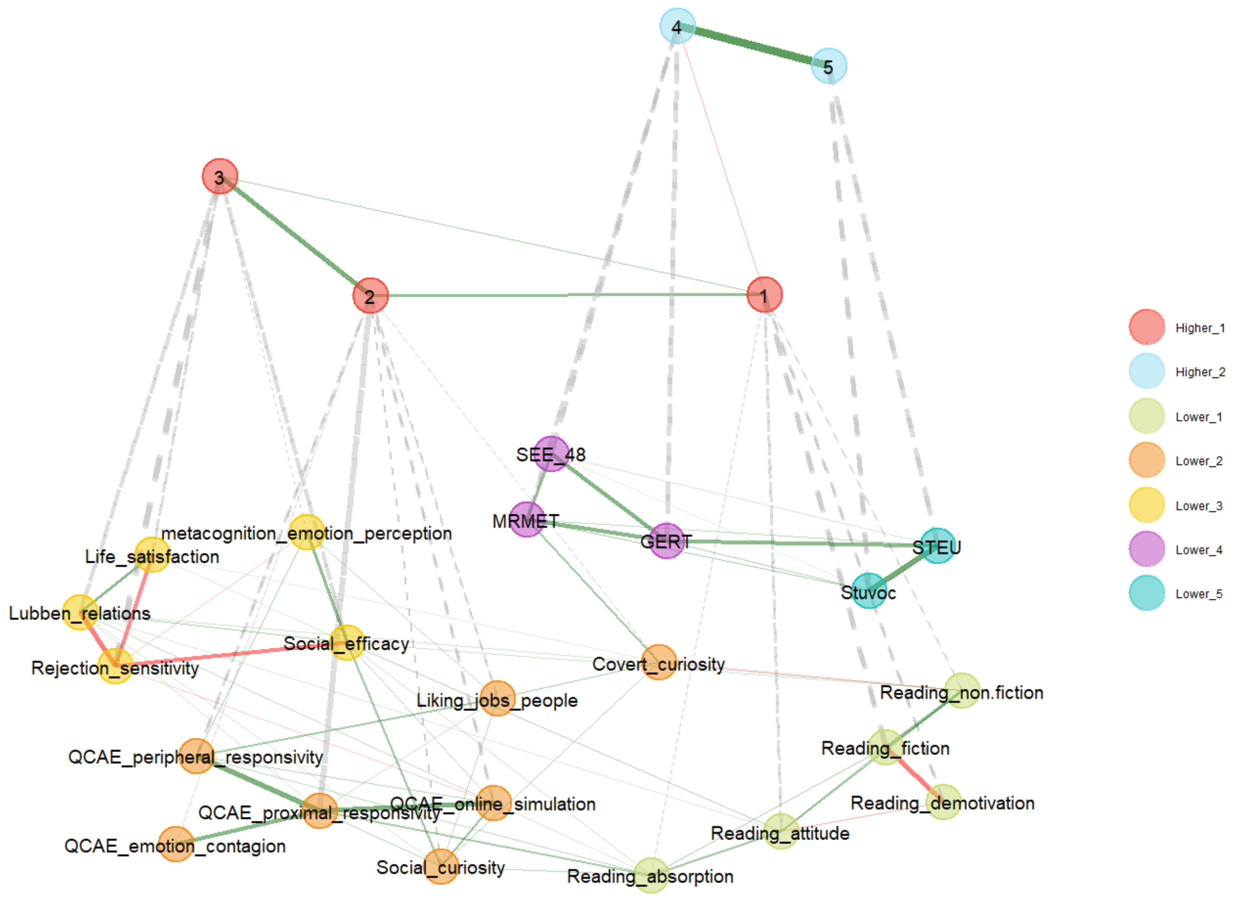

Figure 7: A hierarchical graph analysis when redundant items are dropped.

This analysis also indicates that the cluster of performance-based emotion recognition tests and language tests may be split into two clusters: one containing the emotion recognition tests, and the other containing the language tests and the STEU (the latter also keeps a strong remaining correlation with GERT).

Structural equation modeling of the tests as initially planned

We can also analyse the data with a structural equation model. First, we look at the outcome of the analysis on all tests as initially planned (six factors).

```
emo.model <- 'People_things  =~ Liking_jobs_people + Competence_jobs_people +
                              Occupations_people
                    Reading =~ Reading_motivation + Reading_fiction + Reading_non.fiction
          +
                              Reading_demotivation + Reading_attitude + ART
                              Self_ratings =~ QCAE_proximal_responsivity +
QCAE_peripheral_responsivity
                              + QCAE_emotion_contagion + QCAE_online_simulation +
                              Reading_absorption +  QCAE_perspective_taking +
                    Social_confidence
                              + Social_efficacy
                              Performance_emo   =~ STEU + GERT + SEE_48 + MRMET
                              Crystallized_IQ   =~ Stuvoc + GK
                              Social_relations =~ Loneliness + Life_satisfaction +
Lubben_relations +
                              Rejection_concern + Rejection_expectancy + Social_curiosity +
                              Covert_curiosity
             '
fit <- sem(model = emo.model,
          data = Tests_participants,
          estimator= "MLR")
summary(fit, fit.measures=TRUE,standardized=TRUE)
```

Figure 8 shows the outcome. The detailed output can be found in Appendix B.

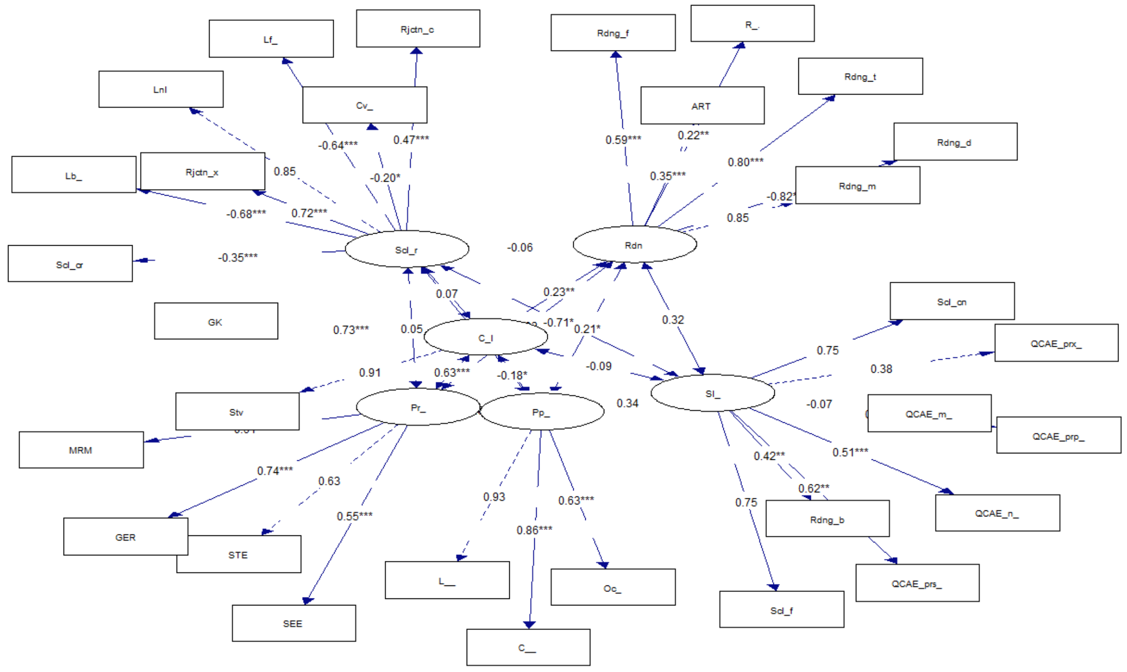

Figure 8: Outcome of a SEM analysis on all tests as initially planned.

The modindices can be used to see how the model can be improved.

modindices(fit, sort = TRUE, maximum.number = 20)

```
                              lhs op                              rhs     mi    epc sepc.lv sepc.all sepc.nox
189                  Crystallized_IQ =~                              ART 79.625  1.076   0.131    0.622    0.622
163                  Performance_emo =~                              ART 54.106  1.450   0.111    0.524    0.524
458   QCAE_proximal_responsivity ~~       QCAE_emotion_contagion 53.503  0.177   0.177    0.494    0.494
457   QCAE_proximal_responsivity ~~ QCAE_peripheral_responsivity 40.978  0.143   0.143    0.437    0.437
481 QCAE_peripheral_responsivity ~~            Social_confidence 38.243 -0.233  -0.233   -0.484   -0.484
153                     Self_ratings =~             Social_curiosity 33.519  1.480   0.348    0.659    0.659
459   QCAE_proximal_responsivity ~~       QCAE_online_simulation 33.393  0.103   0.103    0.406    0.406
462   QCAE_proximal_responsivity ~~            Social_confidence 32.632 -0.214  -0.214   -0.453   -0.453
499       QCAE_emotion_contagion ~~            Social_confidence 31.902 -0.228  -0.228   -0.432   -0.432
562            Social_confidence ~~              Social_efficacy 30.713  0.229   0.229    0.595    0.595
198                  Crystallized_IQ =~                             STEU 24.619  0.478   0.058    0.479    0.479
342                  Reading_fiction ~~          Reading_non.fiction 22.043  0.363   0.363    0.325    0.325
390              Reading_non.fiction ~~             Covert_curiosity 20.710 -0.249  -0.249   -0.304   -0.304
448                              ART ~~                       Stuvoc 18.776  0.005   0.005    0.481    0.481
463   QCAE_proximal_responsivity ~~              Social_efficacy 18.541 -0.089  -0.089   -0.340   -0.340
630                            MRMET ~~             Covert_curiosity 18.350  0.018   0.018    0.306    0.306
477 QCAE_peripheral_responsivity ~~       QCAE_emotion_contagion 17.881  0.104   0.104    0.284    0.284
108                    People_things =~             Covert_curiosity 16.495  0.187   0.194    0.289    0.289
89                     People_things =~ QCAE_peripheral_responsivity 14.796  0.164   0.171    0.280    0.280
149                     Self_ratings =~            Life_satisfaction 13.573 -2.334  -0.549   -0.370   -0.370
```

Structural equation modeling of the nonredundant tests

We can also limit the analysis to the nonredundant tests from the EGA analysis. Reading_attitude turned out to be a better predictor than Reading_demotivation. There were also too high residual correlations between, QCAE_proximal_responsivity and QCAE_emotion_contagion, and between Stuvoc and STEU.

This resulted in the following model:

```
emo.model <- 'Reading =~ Reading_attitude + Reading_fiction
                    Ratings_empathy =~ QCAE_proximal_responsivity +
QCAE_peripheral_responsivity +
                    QCAE_emotion_contagion + QCAE_online_simulation +
            Reading_absorption
                    + metacognition_emotion_perception + Liking_jobs_people +
                    Social_curiosity + Covert_curiosity
                    Performance   =~ GERT + Stuvoc + STEU + SEE_48 + MRMET
                    Social_relations =~ Rejection_expectancy + Life_satisfaction +
Lubben_relations +
                    Rejection_concern + Social_efficacy
                    QCAE_proximal_responsivity ~~ QCAE_emotion_contagion
                    Stuvoc ~~ STEU
                    '

fit <- sem(model = emo.model,
      data = Tests_participants,
      estimator= "MLR")
summary(fit, fit.measures=TRUE,standardized=TRUE)
```

Figure 9 shows a graph (see Appendix C for details).

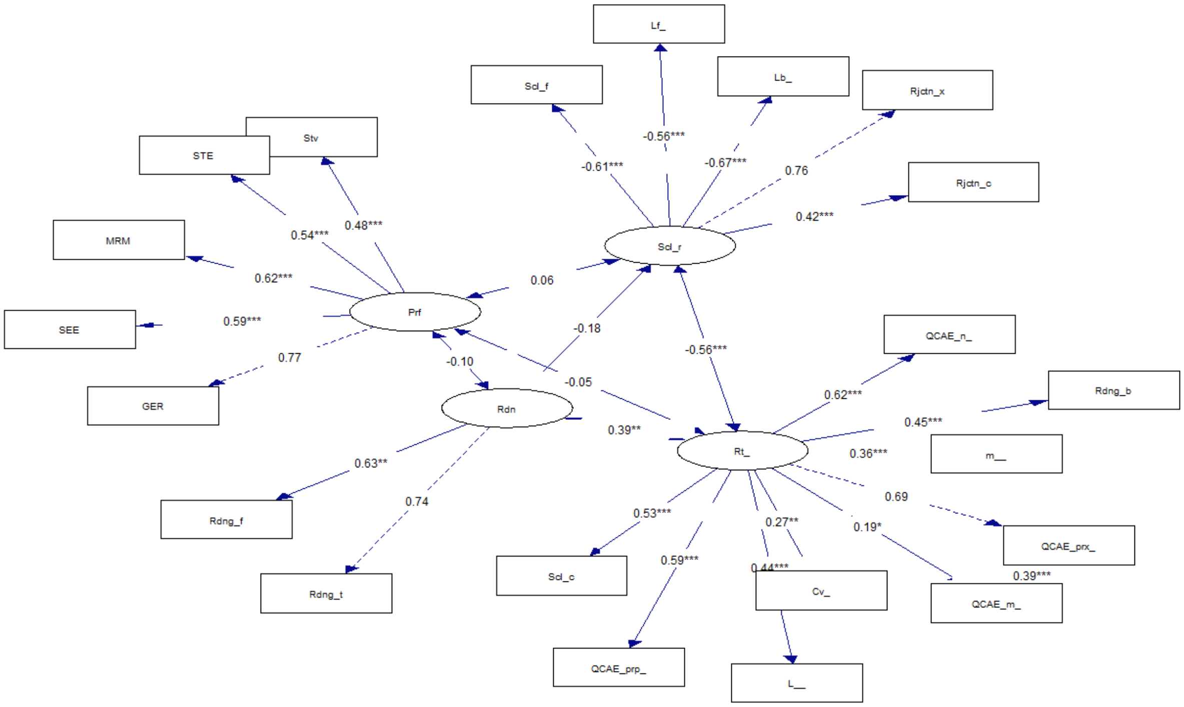

Figure 9: Outcome of a SEM analysis with non-redundant tests.

The modindices indicate how the model could be further improved:

	lhs	op	rhs	mi	epc	sepc.lv	sepc.all	sepc.nox
276	Covert_curiosity	~~	MRMET	17.742	0.017	0.017	0.306	0.306
248	metacognition_emotion_perception	~~	Social_efficacy	16.944	0.084	0.084	0.297	0.297
155	Reading_fiction	~~	Rejection_concern	12.510	0.208	0.208	0.272	0.272
59	Reading	=~	Reading_absorption	11.954	0.310	0.205	0.300	0.300
246	metacognition_emotion_perception	~~	Lubben_relations	11.011	-0.085	-0.085	-0.246	-0.246
64	Reading	=~	GERT	10.816	-0.044	-0.029	-0.234	-0.234
205	QCAE_emotion_contagion	~~	Rejection_concern	9.912	0.109	0.109	0.197	0.197
250	Liking_jobs_people	~~	Covert_curiosity	9.601	0.141	0.141	0.216	0.216
290	GERT	~~	Social_efficacy	9.425	-0.012	-0.012	-0.272	-0.272
82	Ratings_empathy	=~	Life_satisfaction	8.521	-0.932	-0.392	-0.264	-0.264
129	Reading_attitude	~~	Stuvoc	8.186	0.016	0.016	0.234	0.234
96	Performance	=~	Covert_curiosity	8.123	1.472	0.143	0.212	0.212
106	Social_relations	=~	QCAE_emotion_contagion	8.032	0.310	0.165	0.260	0.260
146	Reading_fiction	~~	Covert_curiosity	7.144	-0.114	-0.114	-0.203	-0.203
142	Reading_fiction	~~	Reading_absorption	6.903	0.108	0.108	0.205	0.205
158	QCAE_proximal_responsivity	~~	QCAE_online_simulation	6.842	0.042	0.042	0.227	0.227
261	Social_curiosity	~~	Covert_curiosity	6.793	0.054	0.054	0.186	0.186
179	QCAE_peripheral_responsivity	~~	Social_curiosity	6.741	-0.045	-0.045	-0.204	-0.204
105	Social_relations	=~	QCAE_peripheral_responsivity	6.573	0.264	0.141	0.231	0.231
193	QCAE_emotion_contagion	~~	metacognition_emotion_perception	6.507	-0.051	-0.051	-0.161	-0.161

Conclusion

Figure 1 shows that performance-based emotion recognition tests cluster with tests that typically measure crystallized intelligence, such as vocabulary, general knowledge, and (apparently) knowledge of fiction authors. There is no evidence of clustering with social-emotional functioning, reading motivation, or interest in occupations involving people rather than things. All subsequent analyses support this finding.

Appendix A: Items newly created tests

Table 1

STEU Items used

Item	Question
STEU 2	Xavier completes a difficult task on time and under budget. Xavier is most likely to feel?
STEU 3	An irritating neighbor of Eve's moves to another state. Eve is most likely to feel?
STEU 4	There is great weather on the day Jill is going on an out-door picnic. Jill is most likely to feel?
STEU 8	If the current situation continues, Denise's employer will probably be able to move her job to a location much closer to her home, which she really wants. Denise is most likely to feel?
STEU 9	Song finds out that a friend of hers has borrowed money from others to pay urgent bills, but has in fact used the money for less serious purposes. Song is most likely to feel?
STEU 12	Charles is meeting a friend to see a movie. The friend is very late and they are not in time to make it to the movie. Charles is most likely to feel?
STEU 16	Jim enjoys spending Saturdays playing with his children in the park. This year they have sporting activities on Saturdays and cannot go to the park with him anymore. Jim is most likely to feel?
STEU 17	If all goes well, then it's fairly likely that Derek's house will increase in value. Derek is most likely to feel?
STEU 19	Megan is looking to buy a house. Something happened and she felt regret. What is most likely to have happened?
STEU 20	Mary was working at her desk. Something happened that caused her to feel surprised. What is most likely to have happened?
STEU 22	Someone thinks that another person has deliberately caused something good to happen to them. They are most likely to feel?
STEU 23	Kevin has been working at his current job for a few years. Out of the blue, he finds that he will receive a promotion. Kevin is most likely to feel?
STEU 24	By their own actions, a person reaches a goal they wanted to reach. The person is most likely to feel?
STEU 25	An unwanted situation becomes less likely or stops altogether. The person involved is most likely to feel?
STEU 26	Hasad tries to use his new mobile phone. He has always been able to work out how to use different appliances, but he cannot get the phone to function. Hasad is most likely to feel?
STEU 27	Dorian's friend is ill and coughs all over him without bothering to turn away or cover his mouth. Dorian is most likely to feel?

STEU 29	Quan and his wife are talking about what happened to them that day. Something happened that caused Quan to feel surprised. What is most likely to have happened?
STEU 30	An upcoming event might have bad consequences. Nothing much can be done to alter this. The person involved would be most likely to feel?
STEU 33	A supervisor who is unpleasant to work for leaves Alfonso's work. Alfonso is most likely to<br>feel?
STEU 34	The nature of Sara's job changes due to unpredictable factors and she no longer gets to do the portions of her work that she most enjoyed. Sara is most likely to feel?
STEU 35	Leila has been unable to sleep well lately and there are no changes in her life that might indicate why. Leila is most likely to feel?
STEU 36	A person feels they have control over a situation, but that the reactions of some colleagues complicate the solution. The person involved is most likely to feel?
STEU 37	Someone believes another person has deliberately caused something good to stop happening to them. However, they feel they can do something about it. They are most likely to feel?
STEU 40	Pete gets home late, after his favorite TV show has ended. Pete's partner has taped the show for him. Pete is most likely to feel?
STEU 42	Penny's hockey team trained hard and won the championship. Penny is most likely to feel?

Table 2

Pair	Things-Oriented Occupation	Person-Oriented Occupation
1*	Astronautic engineer	Physician
2	Pharmacist	Dentist
3*	Civil engineer	Lawyer
4	Airplane pilot	Bank manager
5	Chemist	Psychologist
6	Economist	Stock and bond sales agent
7	Architect	Police detective

Occupations Questionnaire Items

8*	Electrical engineer	Registered nurse
9	Mechanical engineer	Fire fighter
10*	Industrial engineer	Real estate agent
11	Biological scientist	Musician
12*	Computer systems analyst	Dietitian
13*	Computer programmer	Property manager
14	Statistician	Editor
15	Accountant	Secondary school teacher
16*	Drafter	Social worker
17*	Clinical Laboratory technologist	Elementary school teacher
18*	Electrician	Clergy
19*	Machinist	Postal clerk
20	Sculptor	Librarian
21	Carpenter	Photographer
22*	Farmer	Secretary
23	Mail carrier, post office	Bank teller
24*	Automobile mechanic	Bookkeeper
25*	Welder	Hairdresser
26*	Painter, construction and maintenance	Receptionist
27	Cook	Bus driver
28*	Roofer	Waiter
29*	Truck driver	Cashier
30	File clerk	Private household servant

Note. We presented 30 item pairs within this questionnaire. Of these pairs, 16 had factor loadings greater than 0.4 and are most interesting for further testing. These pairs are identified with an asterisk.

Table 3

Metacognition Items

Emotion	Area	Item
Pain	Eyes	It is hard for me to tell from someone's eyes if they are in pain.
	Face	It is hard for me to tell from someone's face if they are in pain.
Love	Eyes	It is hard for me to tell from someone's eyes if they are in love.

	Face	It is hard for me to tell from someone's face if they are in love.
Happiness	Eyes	It is hard for me to tell from someone's eyes if they are happy.
	Face	It is hard for me to tell from someone's face if they are happy.
Sadness	Eyes	It is hard for me to tell from someone's eyes if they are sad.
	Face	It is hard for me to tell from someone's face if they are sad.
Surprise	Eyes	It is hard for me to tell from someone's eyes if they are surprised.
	Face	It is hard for me to tell from someone's face if they are surprised.
Disgust	Eyes	It is hard for me to tell from someone's eyes if they are disgusted.
	Face	It is hard for me to tell from someone's face if they are disgusted.
Fear	Eyes	It is hard for me to tell from someone's eyes if they are scared.
	Face	It is hard for me to tell from someone's face if they are scared.
Anger	Eyes	It is hard for me to tell from someone's eyes if they are angry.
	Face	It is hard for me to tell from someone's face if they are angry.

Table 4

PoLR Items

Subscale	Item
Reading Motivation	I read for entertainment
	I read for pleasure
	I read to relax
	I read to become immersed in the world of text
	I read to experience certain emotions
Reading Demotivation	I often don't read because I find it boring
	I often don't read because I never got in the habit
	I often don't read because I don't have to
	I often don't read because I don't see the point
	I often don't read because I'd rather be doing something else
Reading Attitudes	I would like to spend more time reading
	I would like to spend less time reading
	It is important to be well-read
	You can tell a lot about a person by what s/he reads
	I think of myself as a reader
	I feel that what I read is a good reflection of who I am as a person

Table 5

StuVoc4 Items

Word	Extra	Answer	Alternative 1	Alternative 2	Alternative 3
compost	We need some <compost>.	plant material fertilizer	strong support	help to feel better	hard stuff made of stones and sand stuck together
scrumptious	This is <scrumptious>.	delightful in taste	extremely funny	very expensive	very dirty and untidy
authentic	It is <authentic>.	real	noisy	old	deserted
ablution	She performed her <ablutions>.	washed herself to get ready	did her exercises to stay healthy	played her set piece of music	did all her duties as a minister
paltry	This is <paltry>.	not much worth	a group of chickens	a wilderness	notable
amulet	She handled the <amulet>.	silver necklace	cooking tool	delicate situation	spray cleaner
jovial	He was very <jovial>.	friendly	low on the social scale	likely to criticize other	full of himself
ubiquitous	Many unwanted plants are <ubiquitous>.	are found everywhere	are difficult to get rid of	have long, strong roots	die away in the winter
tresses	She hated her <tresses>.	hair	freckles	ankle socks	braces
talon	Just look at those <talons>!	sharp hooks on the feet of a hunting bird	high points of mountains	heavy metal coats to protect against weapons	people who make fools of themselves without realizing it
fracture	They found a <fracture>.	break	small piece	short coat	discount certificate
pragmatic	A <pragmatic>	sensible	controversi	biased	quick

	conclusion.		al		
pumice	Where is my <pumice>?	stone to polish	bracelet	powder brush	power tool
azalea	This <azalea> is very pretty.	plant with many flowers growing in groups	light natural fabric	long piece of material worn in India	sea shell shaped like a fan
anoint	The <anointing> goes well.	rubbing oil on the face	painting the wall	official enrollment	money transfer
refectory	We met in the <refectory>.	room for eating	office where legal papers can be signed	room for several people to sleep in	room with glass walls for growing plants
marsupial	It is <a marsupial>.	an animal with a pocket for babies	an animal with hard feet	a plant that takes several years to grow	a plant with flowers that turn to face the sun
spangled	Her dress was <spangled>.	covered with small bright decorations	torn into thin strips	made with lots of folds of fabric	ruined by touching something very hot
cranny	Look what we found in the <cranny>!	narrow opening	sale of unwanted objects	space for storing things under the roof of a house	large wooden box
atoll	The <atoll> was beautiful.	low island with sea water in the middle	art created by weaving pictures from fine string	small crown with many valuable stones	place where a river flows through a narrow spot with rocks
desist	To <desist> is to	stop	intend	lose hope	wish
rigmarole	I hate the <rigmarole>.	long, pointless and complicated set of actions	very fast and difficult dance for eight people	funny character in theater	form which must be completed each year for tax purposes

anvil	Where did you place the <anvil>?	a block to hammer metal	a clamp for holding wood	a tool for cutting trees	a container to keep things safe
augur	It <augured> well.	promised good things for the future	agreed with what was expected	had a color that looked good with something else	rang with a clear, beautiful sound
candid	Please <be candid>.	say what you really think	be careful	show sympathy	show fairness to both sides
heyday	The town was in its <heyday>.	at its peak of success	on top of the hill	very wealthy	in a state of sleep
rollick	They were <rollicking>.	having fun in a noisy way	driving fast	staying away from school without being permitted to	sliding on snow using round boards
retro	It had <a retro look>.	the look of something from an earlier time	the look of a piece of modern art	the look of something which has been used a lot	a very fashionable look
homogeneous	A <homogeneous group> passed by.	a group of similar people	a group of noisy people	a small group of people	a group of peole that stand out
dachshund	She loves her <dachshund>.	small dog with short legs and a long back	warm fur hat	thick floor rug with special patterns	old musical instrument with strings
impale	She nearly got <impaled>.	stuck through with a sharp instrument	charged with a serious offence	put in prison	involved in a dispute
dingy	It was a <dingy> place.	dirty-looking	dangerous	delightful	hot, dry
soppy	A <soppy> event.	sentimental	not well organized	disappointing	boring

paradigm	You can use that <paradigm>.	set of forms or assumptions	writing style	restatement of a difficult text in easier words	formula list
compound	They made a new <compound>.	thing made of two or more parts	agreement	group of people forming a business	guess based on past experience
gimmick	That's a good <gimmick>.	attention-getting action or image	thing for standing on to work high above the ground	small thing with pockets for holding money	plan of action
drawer	The <drawer> was empty.	sliding box	place where cars are kept	cupboard to keep things cold	animal house
ambiguity	They did not know what to do with <the ambiguity>.	the uncertainty	the cold-blooded animal	the small bird	the group of refugees
conformity	The <conformity> surprised her.	acceptance	relief	coziness	pain
shudder	The boy <shuddered>.	shook	spoke with a low voice	almost fell	called out loudly
maze	They loved the <maze>.	labyrinth	corn	attention they received	cuddly animal
chuck	To <chuck> is to	throw	laugh quietly	scare someone	decorate
awe	They looked at the mountain in <awe>.	with a feeling of respect	with a worried expression	without interest	in fear
peasantry	He did a lot for the <peasantry>.	working class people	house in the countryside	place of worship	businessmen's club
excrete	This was <excreted> recently.	pushed or sent out	made clear	discovered by a science experiment	put on a list of illegal things

caffeine	This contains a lot of <caffeine>.	something that makes you excited	a substance that makes you sleepy	strings from leaves	ideas that are not correct
bylaw	They made a <bylaw>.	secondary law	publisher’s list of older books	code made of lines, read by machines	law that morally condemns people
veer	The car <veered> off.	changed course	came to a stop	made a loud noise	rapidly increased in speed
speedometer	The <speedometer > stopped working.	thing that measures how fast you go	instrument that shows changes in the weather	thing that measures changes in temperature	instrument that makes a car move faster
rascal	She is such <a rascal> sometimes.	a bad girl	an unbeliever	a dedicated student	a hard worker

Appendix B: Output of the analysis with all tests as initially planned

```
lavaan 0.6-20 ended normally after 186 iterations

  Estimator                                         ML
  Optimization method                           NLMINB
  Number of model parameters                        75

  Number of observations                           227

Model Test User Model:
                                              Standard      Scaled
  Test Statistic                              1111.607    1123.385
  Degrees of freedom                               390         390
  P-value (Chi-square)                           0.000       0.000
  Scaling correction factor                                  0.990
    Yuan-Bentler correction (Mplus variant)

Model Test Baseline Model:

  Test statistic                              3091.396    3015.601
  Degrees of freedom                               435         435
  P-value                                        0.000       0.000
  Scaling correction factor                                  1.025

User Model versus Baseline Model:

  Comparative Fit Index (CFI)                    0.728       0.716
  Tucker-Lewis Index (TLI)                       0.697       0.683

  Robust Comparative Fit Index (CFI)                         0.726
  Robust Tucker-Lewis Index (TLI)                            0.694

Loglikelihood and Information Criteria:

  Loglikelihood user model (H0)              -4232.371   -4232.371
  Scaling correction factor                                  1.197
      for the MLR correction
  Loglikelihood unrestricted model (H1)      -3676.568   -3676.568
  Scaling correction factor                                  1.023
      for the MLR correction

  Akaike (AIC)                                8614.743    8614.743
```

```
  Bayesian (BIC)                              8871.614    8871.614
  Sample-size adjusted Bayesian (SABIC)       8633.918    8633.918

Root Mean Square Error of Approximation:

  RMSEA                                          0.090       0.091
  90 Percent confidence interval - lower         0.084       0.085
  90 Percent confidence interval - upper         0.097       0.097
  P-value H_0: RMSEA <= 0.050                    0.000       0.000
  P-value H_0: RMSEA >= 0.080                    0.997       0.998

  Robust RMSEA                                               0.091
  90 Percent confidence interval - lower                     0.084
  90 Percent confidence interval - upper                     0.097
  P-value H_0: Robust RMSEA <= 0.050                         0.000
  P-value H_0: Robust RMSEA >= 0.080                         0.997

Standardized Root Mean Square Residual:

  SRMR                                           0.107       0.107

Parameter Estimates:

  Standard errors                             Sandwich
  Information bread                           Observed
  Observed information based on                Hessian

Latent Variables:
                      Estimate  Std.Err  z-value  P(>|z|)   Std.lv  Std.all
  People_things =~
    Liking_jbs_ppl       1.000                               1.040    0.926
    Cmptnc_jbs_ppl       0.841    0.077   10.893    0.000    0.874    0.856
    Occupatins_ppl       0.123    0.012   10.246    0.000    0.128    0.633
  Reading =~
    Reading_motvtn       1.000                               1.109    0.853
    Reading_fictin       0.589    0.066    8.866    0.000    0.652    0.587
    Readng_nn.fctn       0.259    0.100    2.595    0.009    0.287    0.225
    Reading_dmtvtn      -1.142    0.068  -16.852    0.000   -1.266   -0.823
    Reading_attitd       0.642    0.056   11.542    0.000    0.711    0.800
    ART                  0.067    0.013    5.099    0.000    0.074    0.351
  Self_ratings =~
    QCAE_prxml_rsp       1.000                               0.235    0.383
    QCAE_prphrl_rs       0.817    0.161    5.067    0.000    0.192    0.315
```

```
    QCAE_mtn_cntgn      -0.202    0.482   -0.419    0.676   -0.047   -0.075
    QCAE_nln_smltn       1.132    0.317    3.574    0.000    0.266    0.510
    Reading_bsrptn       1.217    0.408    2.982    0.003    0.286    0.418
    QCAE_prspctv_t       1.285    0.456    2.817    0.005    0.302    0.622
    Social_confdnc       4.026    2.350    1.713    0.087    0.946    0.751
    Social_efficcy       2.218    1.215    1.825    0.068    0.521    0.748
  Performance_emo =~
    STEU                 1.000                               0.076    0.626
    GERT                 1.221    0.251    4.869    0.000    0.093    0.744
    SEE_48               0.819    0.165    4.953    0.000    0.062    0.547
    MRMET                0.898    0.155    5.792    0.000    0.068    0.609
  Crystallized_IQ =~
    Stuvoc               1.000                               0.122    0.909
    GK                   0.777    0.114    6.835    0.000    0.095    0.726
  Social_relations =~
    Loneliness           1.000                               0.543    0.851
    Life_satisfctn      -1.744    0.154  -11.362    0.000   -0.947   -0.638
    Lubben_relatns      -1.142    0.116   -9.863    0.000   -0.620   -0.682
    Rejectin_cncrn       0.849    0.134    6.313    0.000    0.461    0.472
    Rejctn_xpctncy       0.919    0.086   10.710    0.000    0.499    0.715
    Social_curisty      -0.338    0.087   -3.878    0.000   -0.184   -0.348
    Covert_curisty      -0.243    0.104   -2.326    0.020   -0.132   -0.196

Covariances:
                    Estimate  Std.Err  z-value  P(>|z|)   Std.lv  Std.all
  People_things ~~
    Reading            0.238    0.093    2.570    0.010    0.207    0.207
    Self_ratings       0.084    0.062    1.346    0.178    0.342    0.342
    Performance_em     0.001    0.006    0.200    0.841    0.016    0.016
    Crystallizd_IQ    -0.022    0.011   -2.069    0.039   -0.176   -0.176
    Social_relatns    -0.134    0.053   -2.522    0.012   -0.238   -0.238
  Reading ~~
    Self_ratings       0.084    0.053    1.593    0.111    0.321    0.321
    Performance_em     0.002    0.009    0.253    0.800    0.027    0.027
    Crystallizd_IQ     0.031    0.011    2.832    0.005    0.229    0.229
    Social_relatns    -0.034    0.047   -0.724    0.469   -0.056   -0.056
  Self_ratings ~~
    Performance_em    -0.003    0.002   -1.947    0.052   -0.191   -0.191
    Crystallizd_IQ    -0.003    0.003   -0.849    0.396   -0.095   -0.095
    Social_relatns    -0.091    0.039   -2.332    0.020   -0.713   -0.713
  Performance_emo ~~
    Crystallizd_IQ     0.006    0.002    3.467    0.001    0.626    0.626
    Social_relatns     0.002    0.003    0.616    0.538    0.052    0.052
```

```
  Crystallized_IQ ~~
    Social_relatns      0.005    0.005    0.906    0.365    0.068    0.068

Variances:
                   Estimate  Std.Err  z-value  P(>|z|)   Std.lv  Std.all
   .Liking_jbs_ppl    0.178    0.066    2.687    0.007    0.178    0.142
   .Cmptnc_jbs_ppl    0.279    0.080    3.472    0.001    0.279    0.268
   .Occupatins_ppl    0.024    0.002   10.986    0.000    0.024    0.600
   .Reading_motvtn    0.461    0.088    5.238    0.000    0.461    0.273
   .Reading_fictin    0.808    0.078   10.366    0.000    0.808    0.655
   .Readng_nn.fctn    1.546    0.144   10.702    0.000    1.546    0.949
   .Reading_dmtvtn    0.762    0.110    6.957    0.000    0.762    0.322
   .Reading_attitd    0.285    0.039    7.381    0.000    0.285    0.361
   .ART               0.039    0.004   10.682    0.000    0.039    0.877
   .QCAE_prxml_rsp    0.321    0.046    6.928    0.000    0.321    0.853
   .QCAE_prphrl_rs    0.335    0.035    9.702    0.000    0.335    0.901
   .QCAE_mtn_cntgn    0.401    0.038   10.571    0.000    0.401    0.994
   .QCAE_nln_smltn    0.201    0.031    6.405    0.000    0.201    0.740
   .Reading_bsrptn    0.387    0.038   10.061    0.000    0.387    0.825
   .QCAE_prspctv_t    0.145    0.022    6.530    0.000    0.145    0.613
   .Social_confdnc    0.692    0.220    3.143    0.002    0.692    0.436
   .Social_efficcy    0.214    0.053    4.049    0.000    0.214    0.441
   .STEU              0.009    0.002    6.021    0.000    0.009    0.608
   .GERT              0.007    0.001    5.125    0.000    0.007    0.447
   .SEE_48            0.009    0.001    9.128    0.000    0.009    0.701
   .MRMET             0.008    0.001    9.005    0.000    0.008    0.630
   .Stuvoc            0.003    0.002    1.845    0.065    0.003    0.174
   .GK                0.008    0.001    5.751    0.000    0.008    0.473
   .Loneliness        0.112    0.019    5.805    0.000    0.112    0.275
   .Life_satisfctn    1.306    0.129   10.142    0.000    1.306    0.593
   .Lubben_relatns    0.443    0.053    8.393    0.000    0.443    0.535
   .Rejectin_cncrn    0.738    0.077    9.586    0.000    0.738    0.777
   .Rejctn_xpctncy    0.238    0.033    7.320    0.000    0.238    0.489
   .Social_curisty    0.245    0.025    9.928    0.000    0.245    0.879
   .Covert_curisty    0.435    0.038   11.334    0.000    0.435    0.962
    People_things     1.081    0.132    8.177    0.000    1.000    1.000
    Reading           1.229    0.218    5.642    0.000    1.000    1.000
    Self_ratings      0.055    0.050    1.108    0.268    1.000    1.000
    Performance_em    0.006    0.002    3.721    0.000    1.000    1.000
    Crystallizd_IQ    0.015    0.003    5.361    0.000    1.000    1.000
    Social_relatns    0.295    0.034    8.714    0.000    1.000    1.000
```

Appendix C

Output of the analysis when taking into account local dependencies

```
lavaan 0.6-20 ended normally after 102 iterations

  Estimator                                         ML
  Optimization method                           NLMINB
  Number of model parameters                        50

  Number of observations                           227

Model Test User Model:
                                              Standard      Scaled
  Test Statistic                               390.465     383.787
  Degrees of freedom                               181         181
  P-value (Chi-square)                           0.000       0.000
  Scaling correction factor                                  1.017
    Yuan-Bentler correction (Mplus variant)

Model Test Baseline Model:

  Test statistic                              1296.232    1248.839
  Degrees of freedom                               210         210
  P-value                                        0.000       0.000
  Scaling correction factor                                  1.038

User Model versus Baseline Model:

  Comparative Fit Index (CFI)                    0.807       0.805
  Tucker-Lewis Index (TLI)                       0.776       0.774

  Robust Comparative Fit Index (CFI)                         0.809
  Robust Tucker-Lewis Index (TLI)                            0.778

Loglikelihood and Information Criteria:

  Loglikelihood user model (H0)              -2907.000   -2907.000
  Scaling correction factor                                  1.090
      for the MLR correction
  Loglikelihood unrestricted model (H1)      -2711.767   -2711.767
  Scaling correction factor                                  1.033
      for the MLR correction

  Akaike (AIC)                                5913.999    5913.999
  Bayesian (BIC)                              6085.247    6085.247
  Sample-size adjusted Bayesian (SABIC)       5926.782    5926.782

Root Mean Square Error of Approximation:

  RMSEA                                          0.071       0.070
  90 Percent confidence interval - lower         0.062       0.061
  90 Percent confidence interval - upper         0.081       0.080
  P-value H_0: RMSEA <= 0.050                    0.000       0.000
```

```
  P-value H_0: RMSEA >= 0.080                        0.073       0.049

  Robust RMSEA                                                   0.071
  90 Percent confidence interval - lower                         0.061
  90 Percent confidence interval - upper                         0.081
  P-value H_0: Robust RMSEA <= 0.050                             0.000
  P-value H_0: Robust RMSEA >= 0.080                             0.064

Standardized Root Mean Square Residual:

  SRMR                                               0.080       0.080

Parameter Estimates:

  Standard errors                                 Sandwich
  Information bread                               Observed
  Observed information based on                    Hessian

Latent Variables:
                      Estimate  Std.Err  z-value  P(>|z|)   Std.lv  Std.all
  Reading =~
    Reading_attitd       1.000                               0.661    0.743
    Reading_fictin       1.057    0.326    3.242    0.001    0.699    0.629
  Ratings_empathy =~
    QCAE_prxml_rsp       1.000                               0.421    0.686
    QCAE_prphrl_rs       0.849    0.099    8.574    0.000    0.357    0.586
    QCAE_mtn_cntgn       0.286    0.127    2.257    0.024    0.120    0.189
    QCAE_nln_smltn       0.766    0.102    7.531    0.000    0.322    0.619
    Reading_bsrptn       0.737    0.135    5.443    0.000    0.310    0.453
    mtcgntn_mtn_pr       0.470    0.103    4.568    0.000    0.198    0.363
    Liking_jbs_ppl       1.181    0.233    5.064    0.000    0.497    0.443
    Social_curisty       0.660    0.134    4.936    0.000    0.278    0.526
    Covert_curisty       0.424    0.160    2.655    0.008    0.178    0.265
  Performance =~
    GERT                 1.000                               0.097    0.773
    Stuvoc               0.668    0.152    4.393    0.000    0.065    0.481
    STEU                 0.686    0.120    5.715    0.000    0.066    0.545
    SEE_48               0.697    0.106    6.547    0.000    0.067    0.591
    MRMET                0.722    0.117    6.165    0.000    0.070    0.621
  Social_relations =~
    Rejctn_xpctncy       1.000                               0.533    0.764
    Life_satisfctn      -1.567    0.195   -8.058    0.000   -0.836   -0.563
    Lubben_relatns      -1.143    0.154   -7.418    0.000   -0.609   -0.670
    Rejectin_cncrn       0.763    0.170    4.480    0.000    0.407    0.417
    Social_efficcy      -0.795    0.102   -7.768    0.000   -0.424   -0.608

Covariances:
                                Estimate  Std.Err  z-value  P(>|z|)   Std.lv  Std.all
 .QCAE_proximal_responsivity ~~
   .QCAE_mtn_cntgn                 0.109    0.026    4.257    0.000    0.109    0.393
 .Stuvoc ~~
   .STEU                           0.004    0.001    3.836    0.000    0.004    0.362
  Reading ~~
    Ratings_empthy                 0.108    0.035    3.114    0.002    0.387    0.387
    Performance                   -0.006    0.008   -0.782    0.434   -0.096   -0.096
```

```
    Social_relatns            -0.063    0.037   -1.695    0.090   -0.178   -0.178
  Ratings_empathy ~~
    Performance               -0.002    0.004   -0.513    0.608   -0.049   -0.049
    Social_relatns            -0.125    0.021   -5.913    0.000   -0.557   -0.557
  Performance ~~
    Social_relatns             0.003    0.005    0.700    0.484    0.062    0.062

Variances:
                   Estimate  Std.Err  z-value  P(>|z|)   Std.lv  Std.all
   .Reading_attitd    0.354    0.141    2.520    0.012    0.354    0.448
   .Reading_fictin    0.745    0.162    4.590    0.000    0.745    0.604
   .QCAE_prxml_rsp    0.199    0.030    6.637    0.000    0.199    0.529
   .QCAE_prphrl_rs    0.244    0.027    9.047    0.000    0.244    0.656
   .QCAE_mtn_cntgn    0.389    0.037   10.560    0.000    0.389    0.964
   .QCAE_nln_smltn    0.168    0.022    7.496    0.000    0.168    0.617
   .Reading_bsrptn    0.372    0.034   11.085    0.000    0.372    0.795
   .mtcgntn_mtn_pr    0.259    0.029    8.837    0.000    0.259    0.869
   .Liking_jbs_ppl    1.012    0.126    8.008    0.000    1.012    0.804
   .Social_curisty    0.201    0.024    8.244    0.000    0.201    0.723
   .Covert_curisty    0.420    0.039   10.775    0.000    0.420    0.930
   .GERT              0.006    0.001    4.719    0.000    0.006    0.403
   .Stuvoc            0.014    0.002    8.826    0.000    0.014    0.768
   .STEU              0.010    0.001    7.723    0.000    0.010    0.703
   .SEE_48            0.008    0.001    8.675    0.000    0.008    0.651
   .MRMET             0.008    0.001    7.794    0.000    0.008    0.614
   .Rejctn_xpctncy    0.203    0.040    5.089    0.000    0.203    0.417
   .Life_satisfctn    1.504    0.145   10.352    0.000    1.504    0.683
   .Lubben_relatns    0.455    0.061    7.416    0.000    0.455    0.551
   .Rejectin_cncrn    0.785    0.084    9.401    0.000    0.785    0.826
   .Social_efficcy    0.306    0.036    8.444    0.000    0.306    0.631
    Reading           0.437    0.148    2.964    0.003    1.000    1.000
    Ratings_empthy    0.177    0.039    4.535    0.000    1.000    1.000
    Performance       0.009    0.002    5.232    0.000    1.000    1.000
    Social_relatns    0.284    0.049    5.853    0.000    1.000    1.000
```

Appendix D

Full correlation matrix

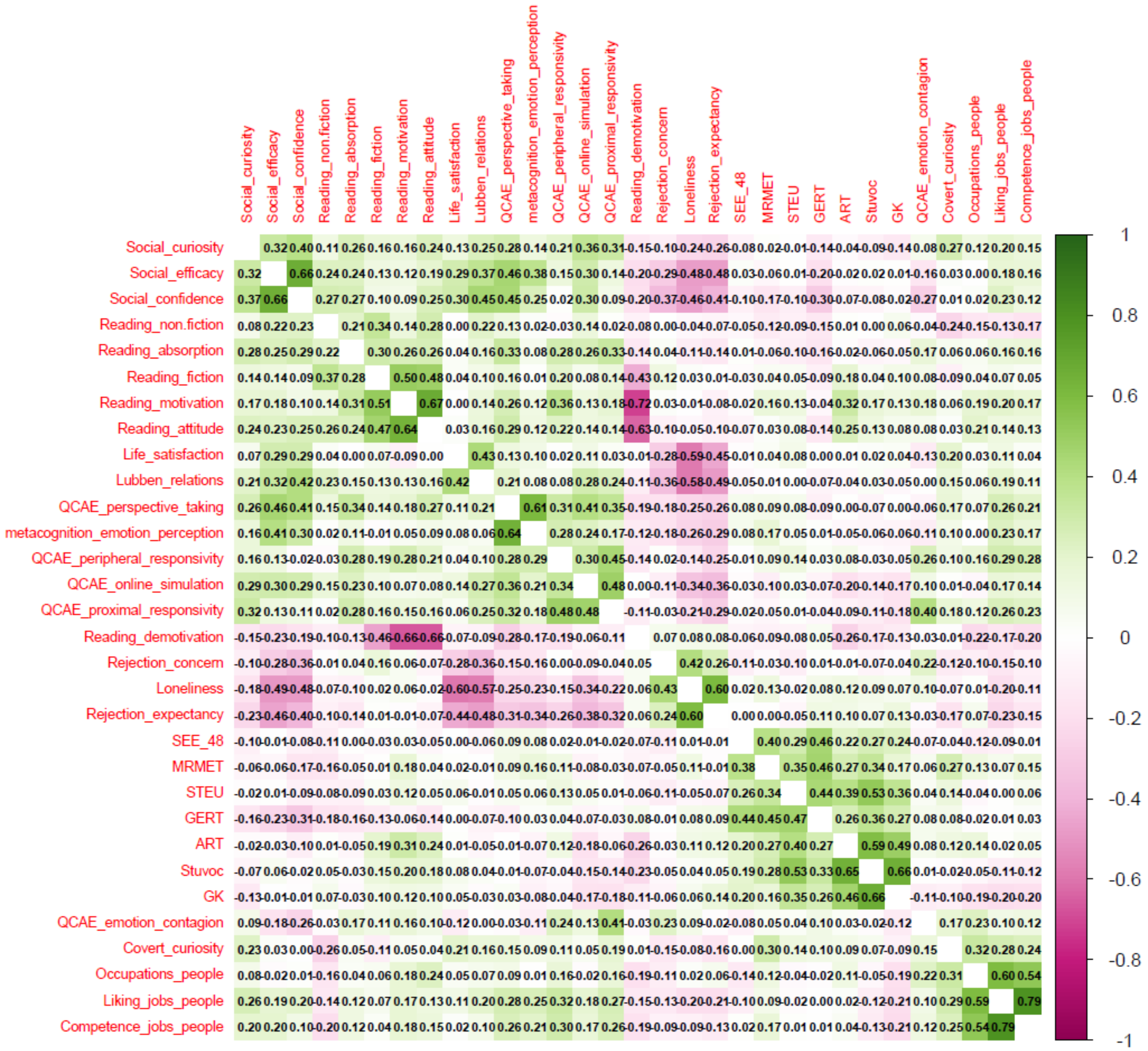

Supplement: Supplementary Materials. — The Supplementary Materials file contains more detailed information about the tests that were used and step-by-step code for the analyses that were run in this article. [file pb-66-1-1443-s1.pdf]
